# Cell Disruption and RNA Extraction from *Rhodococcus* and *Gordonia* Bacteria Grown in the Presence of Hydrophobic Organic Compounds and Heavy Metal Salts

**DOI:** 10.3390/ijms27177890

**Published:** 2026-09-04

**Authors:** Anastasiia V. Krivoruchko, Liliia P. Komarova, Polina Y. Maltseva, Semyon M. Tyan, Anastasia A. Golysheva, Natalia A. Plotnitskaya, Maria S. Kuyukina, Irina B. Ivshina

**Affiliations:** 1 Institute of Ecology and Genetics of Microorganisms of the Ural Branch of the Russian Academy of Sciences, Perm Federal Research Center of the Ural Branch of the Russian Academy of Sciences, 13 Golev Street., 614081 Perm, Russia; dulika5365@gmail.com (L.P.K.); inbox.98@bk.ru (P.Y.M.); vviolent00@mail.ru (S.M.T.); nastay133@gmail.com (A.A.G.); luchnikova.n@mail.ru (N.A.P.); kuyukina@iegm.ru (M.S.K.); 2 Microbiology and Immunology Department, Perm State University, 15 Bukirev Str., 614990 Perm, Russia

**Keywords:** *Rhodococcus*, *Gordonia*, RNA extraction, cell lysis, lysozyme, RNA Solo Kit, complex lipophilic organic compounds

## Abstract

Non-pathogenic actinomycetes play a key role in the degradation of xenobiotics and the biotransformation of complex organic compounds. Studying gene expression in these bacteria can be challenging due to the rigid cell wall that hinders RNA extraction. Here we present the first comprehensive study of total RNA isolation from *Rhodococcus* and *Gordonia* genera, encompassing 21 strains grown under 34 conditions, including *n*-alkanes C_3_–C_16_, terpenoids, non-steroidal anti-inflammatory drugs, used frying oil, and mercury chloride. We compared no lysis strategy and three cell lysis approaches (freezing-thawing, bead beating, and lysozyme treatment) combined with the TRIzol or silica column-based RNA extraction kits. Bead beating, despite aggressive mechanical action, failed to reliably yield intact RNA. We show for the first time that 40 mg/mL of lysozyme with prolonged exposure (1 h) reliably lyses *Rhodococcus* and *Gordonia* cells (96–100% successful isolations), yielding pure integral RNA (118.5–485.8 ng/µL) with the RNA Solo Kit, without requiring detergents. We also developed the first protocol for RNA extraction from *Rhodococcus* cells grown on used frying oil, requiring pre-washing with 0.1 vol.% Tween 80 to disintegrate hard-to-harvest aggregates. These findings establish a robust strategy for RNA isolation from hydrophobic *Rhodococcus* and *Gordonia* bacteria.

## 1. Introduction

Non-pathogenic nocardioform actinomycetes (representatives of the *Bacteria* domain, the *Bacillati* kingdom, the *Actinomycetota* phylum, the *Actinomycetes* class, the *Mycobacteriales* order, particularly the *Corynebacteriaceae*, *Dietziaceae*, *Gordoniaceae* and *Nocardiaceae* families, and particularly the *Corynebacterium*, *Dietzia*, *Gordonia*, *Nocardia* and *Rhodococcus* genera) are degraders of xenobiotics and promising agents for the directed biotransformation of organic compounds. The *Rhodococcus* and *Gordonia* genera are the most promising for bioremediation and biotechnologically important oxidations of target compounds. They utilize a wide range of aromatic compounds [1], *n*-alkanes C_3_–C_31_ [2,3], diesel fuel [4], solvents [5,6] and pharma pollutants originating from widely used non-steroidal anti-inflammatory drugs (NSAIDs) [7,8]. They also accumulate heavy metals [9] and transform terpenoids [10,11] and organic sulfides [12] into biologically active intermediates.

These bacteria have a thick, lipophilic cell wall saturated with mycolic acids and are often cultivated in the presence of hydrophobic substrates and emergent pollutants [13,14,15,16]. In their presence, cells undergo significant changes, including further thickening of the cell wall, production of extracellular polymeric substances (EPS), increased hydrophobicity, shifted zeta-potential, elevated cell surface roughness, and enhanced synthesis of lipids, mycolic acids, and glycolipid biosurfactants [2,3,5,8,10,17]. Understanding how *Rhodococcus* and *Gordonia* regulate degradation pathways and remodel their envelope in response to hydrophobic and toxic substrates requires characterization of their molecular responses (changes in gene expression levels) under such conditions.

Common methods to measure and compare gene expression under different growth conditions are RNA-Seq (transcriptomic analysis) and quantitative PCR with reverse transcription (RT-qPCR) for single genes; both require extraction of intact total RNA from bacterial cells. RNA extraction relies on a chaotropic agent—most often guanidinium thiocyanate—that disrupts membranes and denatures proteins, including immediate inactivation of RNases [18,19,20,21]. Classical TRIzol-based protocols exploit a two-phase water-organic system in which RNA partitions into the upper aqueous phase, away from DNA, proteins and lipids in the interphase and phenol-chloroform phase [18,22,23]; silica-column kits instead avoid phenol and chloroform, precipitating DNA and protein in a high-salt buffer and binding RNA to a silica matrix for elution [20,24].

These protocols are typically developed for lysates, homogenates and whole cells that are readily lysed by guanidinium thiocyanate solutions, but many bacterial cells resist such lysis, and these failures are rarely reported. Specific protocols have therefore been developed for resistant vegetative cells of Gram-positive bacteria such as *Bacillus cereus*, *Enterococcus faecalis*, *Staphylococcus* spp., *Streptococcus* spp. and fast-acid *Mycobacterium smegmatis* [23,25], for *Bacillus subtilis* spores [24], for polysaccharide-rich biofilms of mucoid *Escherichia coli*, *Staphylococcus aureus*, *Pseudomonas aeruginosa* and *Pseudomonas syringae* [26,27], for low-biomass ammonium-oxidizing *Nitrosomonas europaea* and nitrite-oxidizing *Nitrobacter winogradskyi* [21], for intracellular *S. aureus* [28], and for *Streptococcus agalactiae* in infected mouse peritoneal lavage fluid [29]. The disruptors typically used for such refractory prokaryotes are lysozyme, bead beating, detergents, and hot phenol; membranolytic proteins such as porcine myeloid antimicrobial peptide 36 [25] and the polysaccharide lyase Smlt1473 [27] have also been used to break down protective EPS matrices before lysis. In *Rhodococcus* and *Gordonia*, the mycolic acid-rich cell wall is itself a physical barrier to lysis, and this barrier is reinforced further by the very cell-wall thickening, EPS production and lipid enrichment that these bacteria mount in response to hydrophobic and toxic growth substrates [2,3,5,8,10,17]—meaning the adaptations that make them biotechnologically valuable also make their RNA more difficult to extract.

Beyond hindering lysis, growth-medium and environmental components such as organic contaminants, heavy metals, nanoparticles, humic substances, formaldehyde and (poly)phenolic compounds can directly compromise RNA integrity once it is released from the cell, inhibiting downstream PCR enzymes, accelerating degradation, or binding RNA and causing molecular aggregation [30,31,32,33,34,35,36]. Paraffins, for example, stimulate the formation of irreversible RNA adducts [31], and carbon nanotubes distort nucleic acid conformation through π–π stacking interactions [37]. Heavy metals generate reactive oxygen species that damage RNA via 7,8-dihydro-8-oxoguanine formation, metal–RNA adducts, apurinic/apyrimidinic sites and strand breaks [35]; lead and copper catalyze hydrolysis of single-stranded RNA [36], while mercury can directly accelerate RNA cleavage and isomerization [38]. These interactions are especially relevant to gene-expression studies of microbial biodegradation and bioremediation, where the pollutants under investigation may themselves degrade the RNA needed to study the degradation process.

In our routine laboratory practice, we frequently encounter failed or damaged RNA extraction from environmental actinomycetes grown in the presence of contaminants, or while oxidizing recalcitrant hydrophobic compounds, using standard kit protocols or published recommendations for these bacteria [30,39,40,41,42,43,44,45,46,47,48,49,50,51,52,53,54]. The protocols reported for isolating total RNA from *Rhodococcus* and *Gordonia* cells for RT-qPCR and RNA-Seq are summarized in Table S1. Bead beating was the disruption method most frequently applied; lysozyme treatment [39], boiling in 1 M NaOH [46], and passage through a microtip in TRIzol reagent [42] were also used, though the disruption method was not always reported. RNA quantity and quality were rarely specified, and failed attempts, technical problems, and the influence of medium components were not typically discussed. Where reported, results were inconsistent: a soil-specific method was developed to remove humic substances before isolating RNA from *Rhodococcus jostii* [30]; lysozyme at 2 mg/mL for 15 min failed to lyse *Rhodococcus erythropolis* [39]; and protocols validated for *Rhodococcus* sp. RHA1 did not yield sufficient RNA for *Gordonia* sp. NB4-1Y transcriptomics [47]. This fragmented, largely undocumented picture reflects a knowledge gap: no systematic, comparative study of RNA isolation from *Rhodococcus* and *Gordonia*, spanning a broad panel of strains and the growth conditions relevant to their biotechnological use, has previously been performed.

The current study aimed to close this gap by developing a robust, reliable, and reproducible method for extracting total RNA from hydrocarbon-oxidizing actinomycetes, and, for the first time, to evaluate this process systematically across a broad panel of strains and growth conditions.

The working hypothesis was following. A thorough mechanical homogenization rapidly and efficiently disrupts the thick, rigid, lipophilic cell wall of *Rhodococcus* and *Gordonia* cells for proper RNA extraction, with probable difficulties under particular growth conditions that will require the tuning of regimes. Components of growth media (we test hydrocarbons, used frying oil, terpenoids, NSAIDs, mercury chloride, and halloysite nanotubes) may result in a decrease in RNA integrity or yield and require detergents for better cell lysis and RNA extraction. Monophasic TRIzol extraction kits are expected to overcome silica column-based kits in RNA extraction efficacy, as they use solvents providing removal of lipids from the cell wall and dissolution of hydrophobic growth substrates.

At the first step, evenness of cell hydrophobicity levels of actinomycete cells depending on growth conditions was estimated. At the second step, three approaches for RNA extraction were compared: no cell lysis/gentle physical cell disruption, the cell disruption using bead beating, and the cell lysis with lysozyme, all three combined with monophasic TRIzol or silica column-based RNA extraction kits. At lysozyme treatment, an extremely high concentration (40 mg/mL) of lysozyme and a long (1 h) exposure of cells to this enzyme were tested for the first time to lyse *Rhodococcus* and *Gordonia* cells before the RNA extraction. A detergent at a high concentration (10% sodium dodecyl sulphate(SDS)) was used alongside lysozyme to solubilize the cell wall lipids and hydrophobic compounds. At the third step, the suitability of the total RNA obtained using the best lysis/extraction combination was tested in RT-qPCR to estimate expression of the target *alkB* gene coded for alkane-1-monooxygenase, which is induced in the presence of alkanes but not sugars [55].

Criteria of the optimal protocol for extraction of RNA from *Rhodococcus* and *Gordonia* cells included: high reproducibility independent on strain and growth conditions, i.e., integral RNA isolation rates ≥ 80%; RNA integrity, i.e., visibility of the 23 and 16S rRNA bands in electropherograms and the 23S:16S rRNA band ratio ≈ 1.5 or higher; RNA purity, i.e., A260/280 > 1.8 and A260/230 ≥ 2.0; RNA yield ≥ 10 ng/μL; no residual DNA in the total RNA preparation; and suitability of RNA for RT-qPCR.

## 2. Results

### 2.1. Estimation of Physical-Chemical Characteristics of Rhodococcus and Gordonia Cells Before RNA Extraction

**Figure 1:**
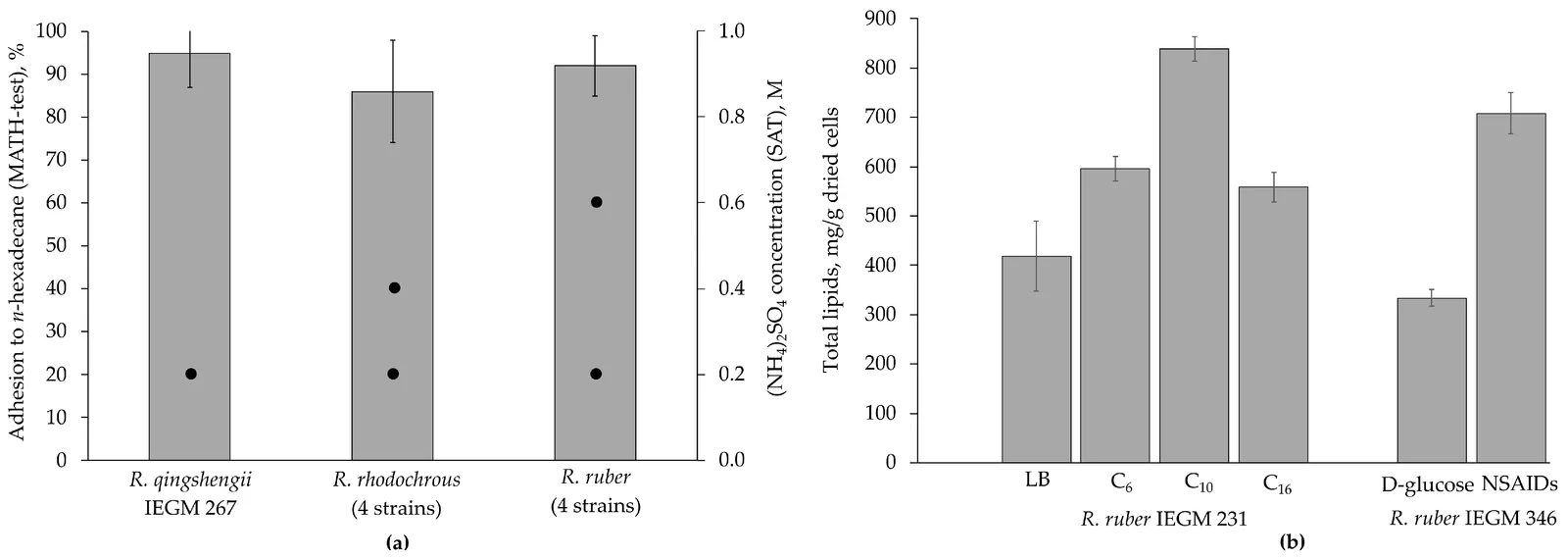
Hydrophobicity and total cellular lipids of *Rhodococcus* strains. (**a**) Hydrophobicity of cells determined by the MATH test (diagram) and Salt Aggregation Test (SAT, black circles). *R. qingshengii* IEGM 267 and *R. rhodochrous* were grown in LB, and all growth conditions for all *R. ruber* strains were summarized. *R. ruber* IEGM 1122 was not included in the analysis of *R. ruber* strains (see Table S2). The minimal (NH_4_)_2_SO_4_ concentrations at which cells aggregate are shown. (**b**) Total lipids extracted from *R. ruber* IEGM 231 and IEGM 346 cells grown on different substrates. C_6_, C_10_, C_16_ refer to *n*-hexane, *n*-decane, and *n*-hexadecane, respectively. The NSAID mixture contains diclofenac, ibuprofen, and naproxen (0.01 g/L each). Growth conditions are detailed in Table S3 and Materials and Methods Section. Means ± standard deviations are shown in (**a**,**b**). Three to six biological replicates were used for each strain, growth condition, and type of analysis.

Before testing various RNA extraction approaches, evenness of *Rhodococcus* and *Gordonia* cells in terms of their physical-chemical characteristics and robustness to lysis was estimated for reference strains and growth conditions. *Rhodococcus* bacteria were shown to be highly hydrophobic, even when grown in Luria-Bertani broth (LB). Regardless of the growth conditions, rhodococci strongly adhered to *n*-hexadecane in the Microbial Adhesion to Hydrocarbons (MATH) test, with 74–100% adhered cells and 91% adhered cells on average, and the cell aggregation occurred at low ammonium sulphate concentrations of 0.2–0.6 M (Figure 1a). As demonstrated with *R. ruber*, neither D-glucose (a water-soluble substrate) nor *n*-alkanes (hydrophobic substrates), added to a mineral medium *Rhodococcus*-surfactant (RS), affected cell surface hydrophobicity. Similarly, terpenoids and NSAIDs appeared to have no significant effect on this parameter (Figure 1a). These compounds also did not alter the zeta potential—an additional parameter to indicate the level of cell hydrophobicity—of *R. ruber* and *R. rhodochrous* cells (Table 1).

While all the *Rhodococcus* strains studied were hydrophobic, *R. cerastii* IEGM 1243 and *R. qingshengii* IEGM 267 appeared to be the most hydrophobic. The zeta potential of these strains grown in LB was 5–11 mV less negative and shifted toward a neutral position compared to the *R. rhodochrous* and *R. ruber* strains (Table 1). Additionally, for *R. qingshengii* IEGM 267, adhesion of this strain to *n*-hexadecane in the MATH test was high (95%), and the (NH_4_)_2_SO_4_ concentration triggering cell aggregation was lowest (0.2 M) (Figure 1a). Moreover, *R. qingshengii* IEGM 267 was just one strain, whose hydrophobic properties were further enhanced in the presence of hydrocarbons. The zeta potential shifted to the neutral position at 11 mV when the IEGM 267 was grown in RS with *n*-hexadecane (Table 1). The detected zeta potential change may be more related to the influence of RS than *n*-hexadecane. LB and RS had different ionic strengths (I, mol/L). This parameter can be calculated as I = ½ Σ c_i_z_i_^2^, where c_i_—concentration of ion i and z_i_—charge of ion i. For LB, I ≈ 0.171 mol/L due to the significant contribution of 10 g/L NaCl and the minimal contribution of tryptone and yeast extract. For RS, I = 0.129 mol/L, which indicates fewer charged ions on the cell surface. We suppose that the influence of the medium is detected in cultures with cells that are initially less charged, have a lower zeta potential, and therefore are sensitive to ionic strength changes. This effect was not observed in *R. rhodochrous* and *R. ruber* cultures, which had a pronounced zeta potential between −37 mV and −31 mV (Table 1).

*Gordonia* cells appeared to be also highly hydrophobic. *G. alkanivorans* IEGM 1277 had the lowest zeta potential of −6 ± 0 mV and exhibited the greatest sensitivity to the composition of the growth medium. Meloxicam in particular shifted the zeta potential of this strain by almost three times to more negative values (Table 1).

The MATH test, SAT, and zeta potential measurements evaluate cell surface hydrophobicity, which is important for contact with lysis solution components (i.e., lysozyme, SDS, and guanidinium thiocyanate). The cell wall lipids contribute to the hydrophobicity and rigidity of the cell wall, and their amounts are rather important for proper cell disruption. As seen from Figure 1b, total lipids in actinomycete cells were present in big amounts and varied in the range from 334 to 839 mg/g dried cells. Moreover, this parameter depended on growth conditions. *n*-Alkanes and NSAIDs resulted in a 1.3–2.1-fold increase in the total lipids of *Rhodococcus* cells, and this effect was more evident for *n*-decane and NSAIDs (Figure 1b).

We supposed that gentle methods of cell lysis before RNA extraction would be ineffective and that detergents would be required for disruption of *Rhodococcus* and *Gordonia* cells. The *R. cerastii* IEGM 1243, *R. qingshengii* IEGM 267, and *G. alkanivorans* IEGM 1277 strains were expected to be particularly difficult to lyse compared to other strains.

### 2.2. Comparison of Different Approaches for Extracting RNA from Rhodococcus and Gordonia Cells

The success rates of RNA isolation depending on the cell lysis method and RNA extraction kit are shown in Table 2 and confirm that gentle lysis methods are insufficient, and that a lysis step is required to overcome the lipophilic barrier for RNA extraction from *Rhodococcus* and *Gordonia* cells. Using RNA isolation kits directly on cell pellets and cycled freezing-thawing prior to the extraction with kits failed to release RNA in every trial (0/9 samples for each combination, Table 2). Mechanical disruption with metal beads was more effective but still largely unreliable: RNA of any quality was recovered in 17–81% of trials depending on the extraction reagent used, while integral RNA was obtained in only 7–17% of these successful extractions (Table 2). At this step, integral RNA was defined as having relative integrity if the 23S and 16S rRNA bands were visible. The rates of RNA isolation using TRIzol™ LS or RNA Solo Kit were 3–5-fold higher compared to RNeasy at bead beating, but all three extraction reagents did not differ in rates of integral RNA isolation (Table 2).

Enzymatic lysis with lysozyme combined with TRIzol™ LS extraction was also ineffective. Although the efficacy of RNA isolation for this combination was registered at 33%, only three samples were positive, and no RNA was obtained that was preliminarily defined as integral (Table 2). In contrast, lysozyme lysis combined with the silica column-based RNA Solo Kit was markedly and significantly more effective than any bead beating combination, yielding RNA in 97% of samples without SDS (all integral) and 82% of samples with SDS (96% integral). These success rates did not differ significantly between the two lysozyme treatments, indicating that SDS addition was not essential once cells had been sufficiently permeabilized by lysozyme (Table 2).

**Figure 2:**
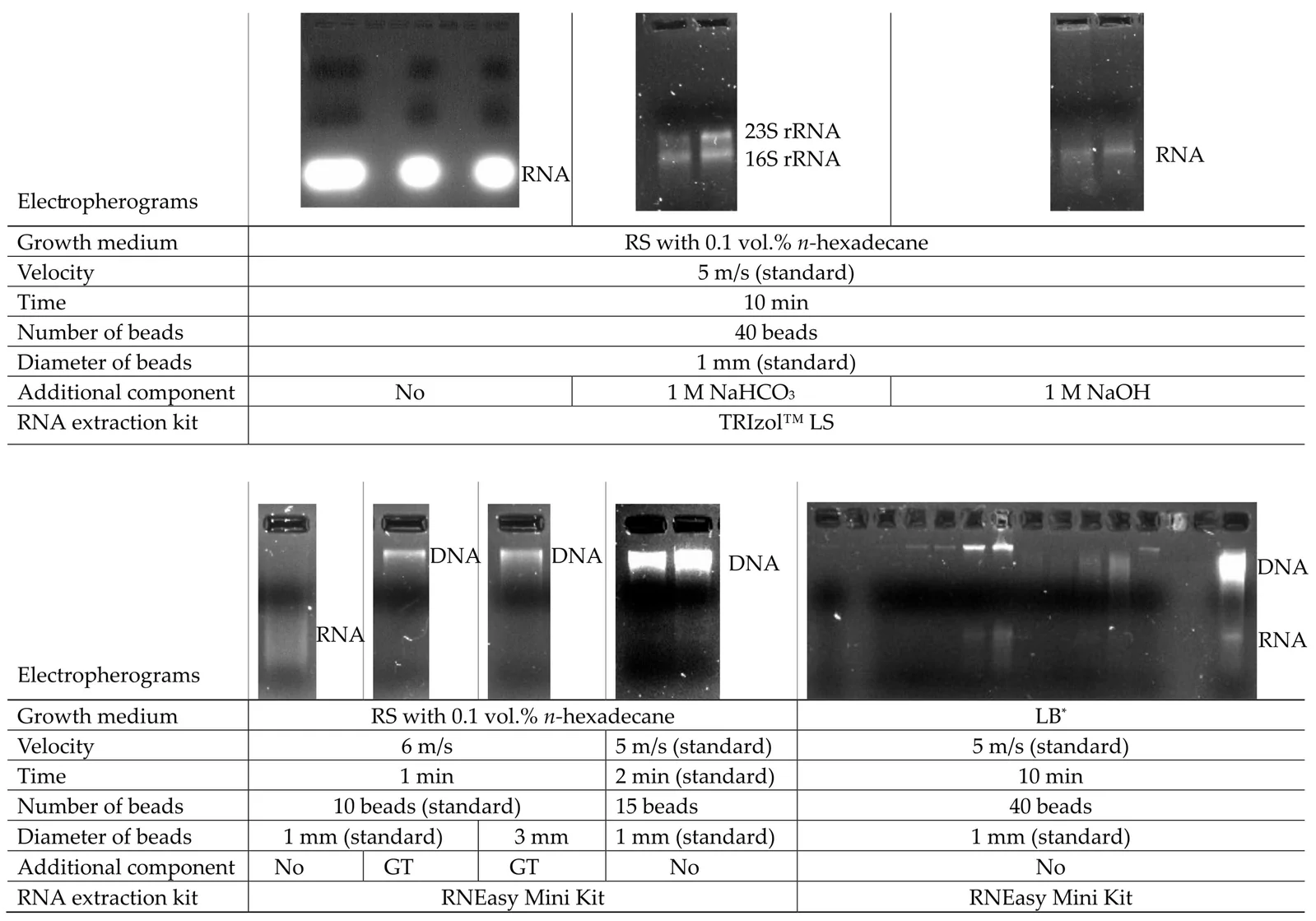
Electropherograms of total RNA obtained after the lysis of *R. ruber* IEGM 231 cells using metal beads. GT—guanidinium thiocyanate. DNase treatment was not performed. * Four biological replicates with 3–4 technical replicates (same sample run in different lanes) were used for this electrophoresis. In other electropherograms, biological replicates are shown.

Modifications to the bead beating protocol—increasing bead number and speed, extending homogenization time, varying bead diameter, and pre-resuspending cells in guanidinium thiocyanate—did not consistently improve RNA recovery, and the RNA obtained was frequently degraded or present as smears rather than distinct bands (Figure 2). Adjusting the lysate pH to 7.0–8.0 with NaHCO_3_ or NaOH prior to phase separation improved the outcome for this method, yielding visible 23S and 16S rRNA bands in place of the diffuse RNA smear obtained without pH adjustment (Figure 2). Although this improvement did not result in an increase in RNA isolation rates.

The integrity of RNA, having relative integrity if the 23S and 16S rRNA bands were visible, was further densitometrically estimated. For this, 23S:16S rRNA ratios were calculated and summarized in Table 3. Where integral RNA was recovered after bead beating, the ratio was highly variable from 0.82 to 2.16, and silica column-based kits recovered 1.4–2.6-fold more integral RNA than TRIzol™ LS. Integrity of RNA obtained after lysozyme lysis was similar to that after bead beating and extraction with silica column-based kits, with a median 23S:16S ratio of 1.38 without SDS and 1.53 with SDS. This difference was not statistically significant, confirming that SDS addition contributed little to lysozyme-mediated cell disruption or the resulting RNA quality. These median ratios are close to, though somewhat below, the value of approximately 1.5 conventionally used as a benchmark for high-quality, non-degraded bacterial RNA [56].

**Figure 3:**
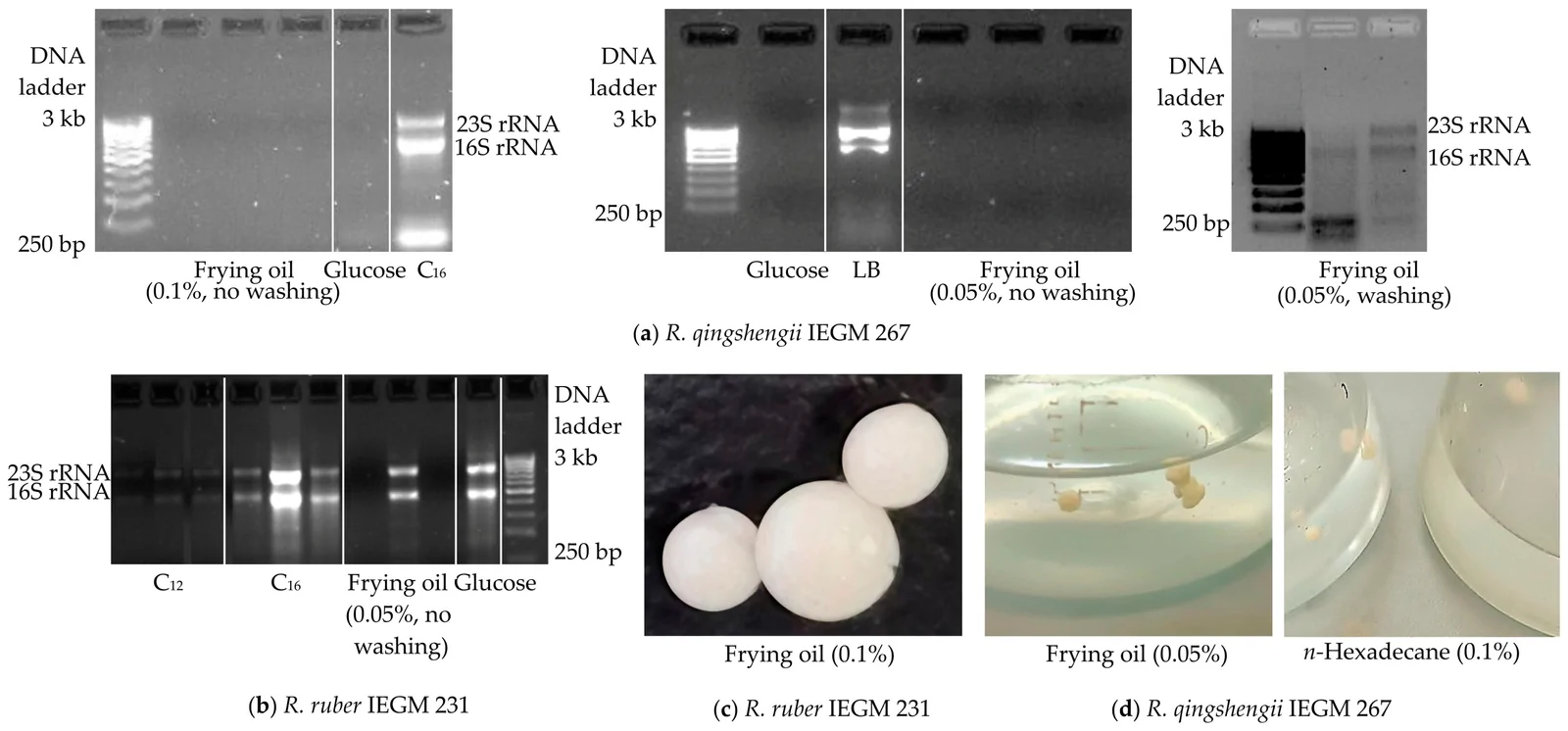
The effects of used frying oil on the growth of *Rhodococcus* strains and the extraction of RNA from these bacteria. (**a**) Electropherograms for RNA recovered from *R. qingshengii* IEGM 267 cells grown in LB and in RS with 1 g/L D-glucose, 0.1 vol.% *n*-hexadecane and either 0.05 or 0.1 vol.% used frying oil. (**b**) Electropherograms for RNA recovered from *R. ruber* IEGM 231 cells grown in RS with 1 g/L D-glucose, 0.1 vol.% *n*-dodecane, *n*-hexadecane or used frying oil. (**c**) Growth of *R. ruber* IEGM 231 cells in RS with 0.1 vol.% used frying oil: The formation of smooth, round aggregates measuring 1–3 mm in diameter can be seen. (**d**) Growth of *R. qingshengii* IEGM 267 cells in RS with 0.05 vol.% used frying oil or 0.1 vol.% *n*-hexadecane: The formation of rather loose, round aggregates measuring 1–3 mm in diameter can be seen. Substrates were added to RS as described in Table S3. C_12_—*n*-dodecane, C_16_—*n*-hexadecane. Cells were lysed using 40 mg/mL lysozyme at 37 °C for 1 h with the addition of 10% SDS. RNA was extracted using RNA Solo Kit with integrated DNase E. “Washing” involved washing the cells with 0.1% Tween 80 and 0.5% NaCl prior to cell lysis. Three biological replicates were used for each strain and growth condition.

The most challenging samples were *Rhodococcus* cells grown in the presence of used frying oil. At 0.05–0.1 vol.% frying oil, *R. qingshengii* IEGM 267 and *R. ruber* IEGM 231 formed dense, smooth, non-adhesive aggregates, or “floating biofilms”, that were difficult to harvest and resistant to disruption (Figure 3). No RNA was recovered from *R. qingshengii* IEGM 267 cells without additional treatment, and RNA was obtained from *R. ruber* IEGM 231 cells just in one sample of three samples. Pre-washing the harvested cell pellets with 0.1% Tween 80 and then with 0.5% NaCl to remove residual oil and disperse aggregates resolved this problem, allowing integral RNA to be recovered from both tested strains (Figure 3). This washing step was not required for cells grown in the presence of liquid *n*-alkanes, which also formed aggregates. These aggregates were softer, fragile, and more easily harvested (Figure 3).

Of the three strains identified in Section 2.1 as likely to be particularly difficult to lyse, this expectation was borne out only for *R. qingshengii* IEGM 267 grown in the presence of used frying oil. The *R. qingshengii* IEGM 267 cells grown under other conditions, as well as *R. cerastii* IEGM 1243 and *G. alkanivorans* IEGM 1277 cells, despite their comparatively hydrophobic and zeta potential-shifted phenotypes (Section 2.1), were reliably lysed by the lysozyme/RNA Solo Kit protocol and consistently yielded RNA under the growth conditions tested.

The effect of growth conditions on RNA isolation outcomes was assessed separately within each lysis method–extraction kit combination. Within the bead beating + RNeasy Mini Kit combination—the most extensively sampled treatment (n = 171 samples across 13 growth-condition categories)—the probability of isolating RNA differed significantly among growth conditions (χ^2^ = 56.0, df = 10, *p* = 0.00). This effect was driven almost entirely by a single condition: cultures grown under a propane:air atmosphere yielded RNA in 59% of samples, compared with 7% across all other conditions combined (Fisher’s exact test, OR = 20.5, *p* = 0.00). No other growth condition tested with this combination—including liquid *n*-alkanes, LB and oligotrophic conditions—exceeded 19% success. However, none of the growth conditions, including gaseous-hydrocarbon growth, resolved into significantly different rates of integral RNA isolation.

**Figure 4:**
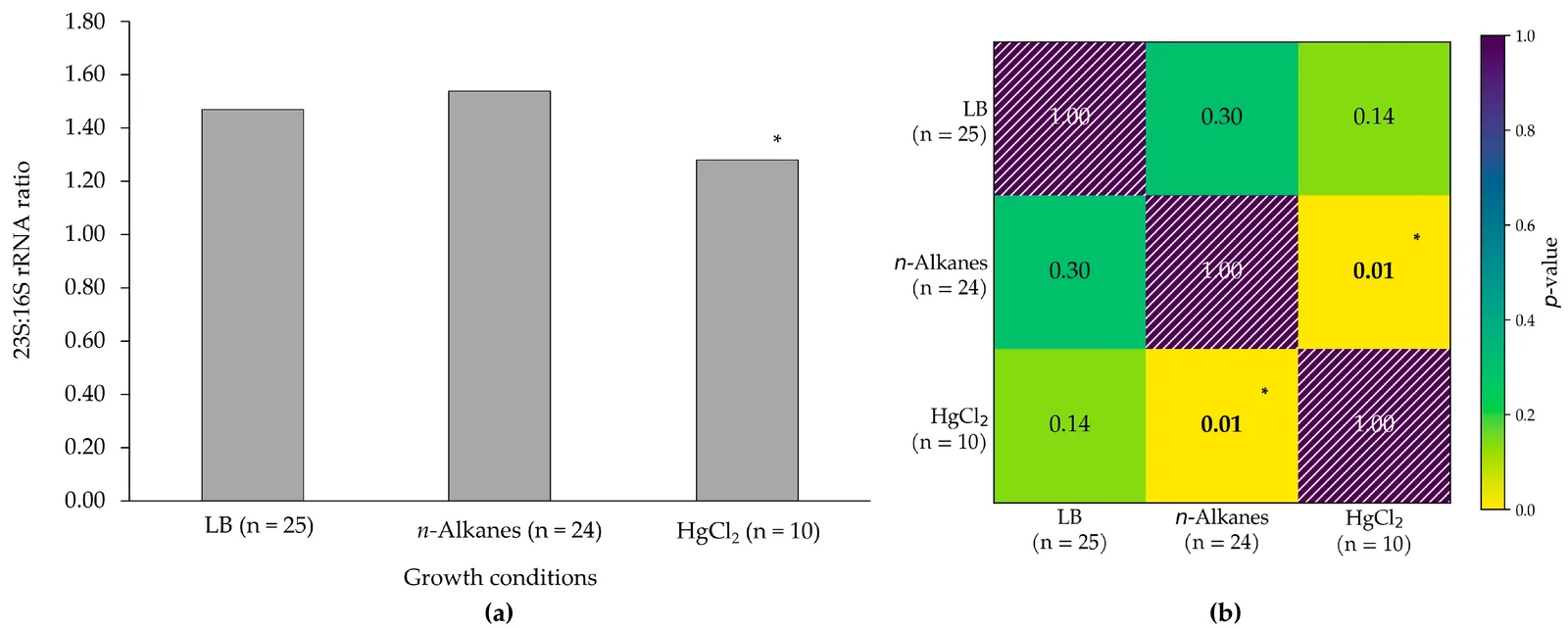
Effects of growth conditions on integrity indices of RNA recovered from *Rhodococcus* and *Gordonia* cells using lysozyme treatment and RNA Solo Kit. (**a**) Median 23S:16S rRNA ratios. (**b**) A heatmap for *p*-values obtained in pairwise comparisons of 23S:16S rRNA ratios in the Mann-Whitney *U* test with the Bonferroni correction. * Difference between HgCl_2_-exposed and *n*-alkane-grown cultures is statistically significant at *p* < 0.05. The n is the number of samples (biological replicates) analyzed.

Within the lysozyme lysis + RNA Solo Kit combination, growth condition had no effect on whether RNA was isolated among the three most extensively tested conditions: LB (n = 27), *n*-alkane (n = 25), and HgCl_2_ (n = 10) exposure each yielded RNA in ≥96% of samples (χ^2^ = 0.40, df = 2, *p* = 0.82). The 23S:16S rRNA ratio also did not vary significantly among these three best-replicated conditions (Kruskal–Wallis test, *H* = 6.2, *p* = 0.05). However, in pairwise comparisons, HgCl_2_-exposed cultures differed from *n*-alkane-grown cultures (Mann–Whitney *U* test with Bonferroni correction, α = 0.0167, *p* = 0.01), with 23S:16S ratios of 0.03–1.41 units lower under HgCl_2_ exposure than under *n*-alkane exposure (Figure 4); neither condition differed significantly from LB. Mercury chloride had a slight negative effect on RNA integrity, enhancing biodegradation of these molecules.

### 2.3. Influence of Lysis Methods and RNA Extraction Kits on RNA Yield and Purity

**Figure 5:**
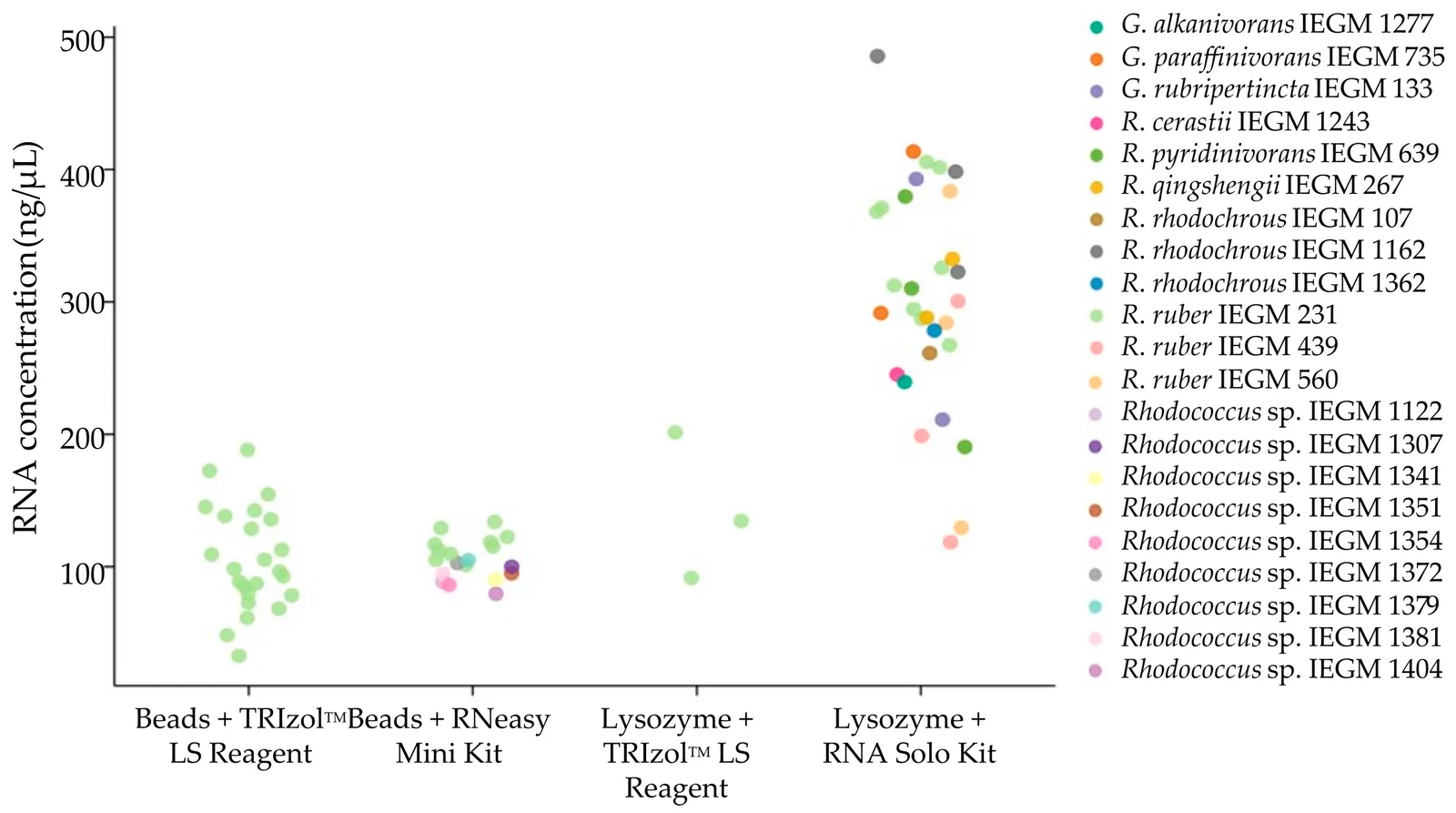
Concentrations of total RNA obtained from *Rhodococcus* and *Gordonia* cells using various lysis methods and RNA extraction kits. Biological replicates with isolated RNA of any quality are shown. Initial amount of biomass used for RNA extraction was 10 ± 1 mg of wet cell weight.

To enable a more accurate comparison of RNA extraction protocols and estimation of the quality of the resulting RNA, the concentration and purity of RNA were measured. The lysis methods used for comparisons were metal bead beating (the standard protocol) and lysozyme treatment (with and without SDS), as these produced the most reliable results. As can be seen in Figure 5, the concentrations of the total RNA recovered varied widely, from 9.8 to 485.8 ng/μL, depending on the lysis method and kit used. On average, lysozyme treatment yielded three times more RNA than bead beating. RNA concentrations in the lysozyme lysates ranged from 91.5 to 485.8 ng/μL, with a median value of 293.1 ng/μL. Meanwhile, total RNA varied from 9.8 to 188.3 ng/μL, with a median value of 101.3 ng/μL, in lysates obtained after bead disruption (Figure 5). The silica column-based kits seemed to be better than the TRIzol method in terms of RNA yield. In combination with metal bead disruption, they produced more consistent RNA concentrations (the difference between the highest and lowest concentrations was no more than twofold) and yielded ≥118.5 ng/μL of RNA in the lysozyme lysates. RNA concentrations differed by a factor of 19 in lysates obtained using the “bead beating + TRIzol” combination, with concentrations not exceeding 201.5 ng/μL in lysates obtained using lysozyme treatment and TRIzol extraction (Figure 5).

The yield values give some insights into the metabolic properties of *Rhodococcus* bacteria. We propose that HgCl_2_ inhibits RNA synthesis (this is a stressful growth condition in the presence of a toxicant) while *n*-alkanes C_3_–C_16_ stimulate this process (degradation of hydrocarbons requires synthesis of oxidative enzymes, mycolic acids and biosurfactants and intense metabolism of fatty acids). The median RNA concentrations for *Rhodococcus* cells grown in LB with and without HgCl_2_ differ significantly at *p* < 0.05 and were 379.8 ng/μL and 247.8 ng/μL, respectively. Meanwhile, the difference between median RNA concentrations for cells grown in RS with *n*-alkanes and glucose was less pronounced but still meaningful (*p* < 0.05). The RNA yields were 369.7 ng/μL and 287.2 ng/μL on *n*-alkanes and D-glucose, respectively.

**Figure 6:**
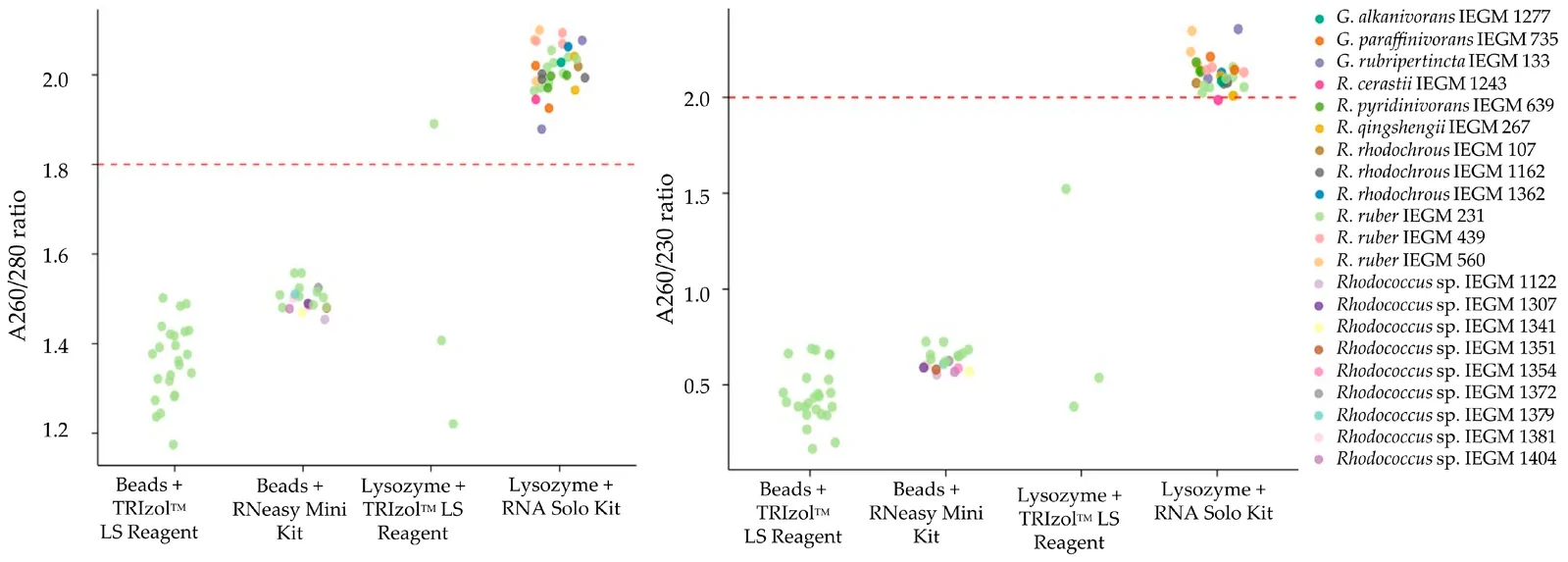
The purity of the total RNA obtained from *Gordonia* and *Rhodococcus* cells using various lysis methods and RNA extraction kits. Purity was determined photometrically by calculating the A260/280 and A260/230 ratios, which indicated the presence of proteins and other components (e.g., phenol, guanidinium thiocyanate, ethanol, and polysaccharides), respectively. The red dashed lines indicate the threshold levels above which the quality of the RNA is appropriate for downstream applications. Biological replicates with isolated RNA of any quality are shown.

Pure RNA was only obtained when lysozyme and the silica column-based RNA Solo Kit were used together. Proteins and other contaminants (e.g., guanidinium thiocyanate, ethanol, and cell polysaccharides) were properly removed, as evidenced by A260/280 > 1.8 and A260/230 ≥ 2.0, respectively. The A260/280 and A260/230 ratios in pure RNA preparations varied from 1.9 to 2.1 and 2.0 to 2.4, respectively (Figure 6). Efficient protein removal was particularly important in the case of lysozyme treatment, given the high concentration of this protein used. Other methods of RNA isolation from *Rhodococcus* and *Gordonia* strains, such as bead beating or the use of TRIzol™ LS in combination with lysozyme cell lysis, did not typically produce RNA of the required purity. The A260/280 and A260/230 ratios in these RNA samples were on average below the threshold level, ranging from 1.2 to 1.9 and from 0.2 to 1.5, respectively (Figure 6).

The RNA yield estimations and RNA purity analysis were concordant with RNA integrity. Samples of total RNA of low integrity (i.e., RNA without distinct 23S and 16S rRNA, or a 23S:16S rRNA ratio of ≤1.00) had an A260/280 ratio of 1.41 ± 0.10, an A260/A230 ratio of 0.50 ± 0.14, and an RNA concentration of 102 ± 37 ng/μL. In contrast, integral RNA (a 23S:16S rRNA ratio of >1.00) had an A260/280 ratio of 1.91 ± 0.21, an A260/A230 ratio of 1.82 ± 0.59, and a concentration of 264 ± 108 ng/μL. These parameters did not affect each other and were not causative; rather, they reflected the efficacy of the RNA extraction. The correlation coefficients (R_Spearman_) between integrity and purity/RNA concentration were 0.71–0.84 (*p* = 0.00), high but below 1.00. Therefore, integral RNA was present in samples with low purity, such as RNA isolated from actinomycete cells using bead beating and RNeasy and having 23S:16S rRNA ratios of 1.70–2.16 (Table 3). Some integral RNA recovered using lysozyme and RNA Solo Kit yielded less than 200 ng/μL of RNA. One of the three successful lysozyme + TRIzol samples yielded 202 ng/μL of RNA, with a purity of 1.9 and 1.5 for A260/280 and A260/A230, respectively. However, the integrity of this RNA was low, with the 23S and 16S rRNA bands not distinguished (Table 2).

### 2.4. Confirmation of RNA Quality in Gene Expression Analysis

The total RNA obtained using lysozyme lysis and the RNA Solo Kit extraction was tested in a gene expression analysis. RNA was isolated from *R. ruber* IEGM 231 cells that were grown in the presence of either 1 g/L glucose or 0.1 vol.% *n*-hexadecane. Alkane-1-monooxygenases AlkB are key enzymes participating in the biodegradation of *n*-alkanes by bacteria [55]. It was expected that levels of *alkB* transcripts would be higher in *Rhodococcus* cells growing on liquid alkanes than on D-glucose. The total RNA obtained was pure, with stable A260/280 and A260/230 ratios of between 2.0 and 2.1. RNA concentrations were 279.8–294.6 ng/μL and 401.7–405.8 ng/μL for cells grown in the presence of D-glucose and *n*-hexadecane, respectively.

**Figure 7:**
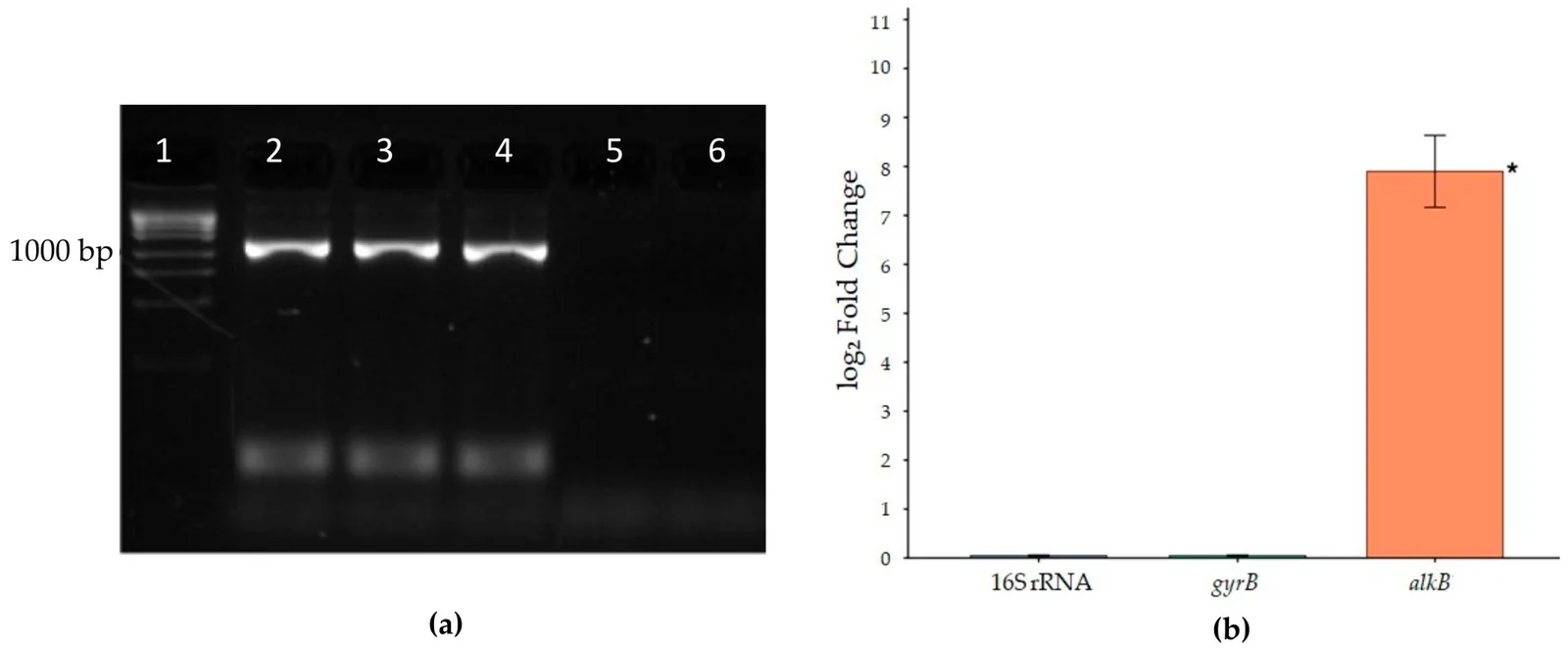
The expression of the *alkB* gene in *R. ruber* IEGM 231 cells grown in the presence of 0.1 vol.% *n*-hexadecane, as determined by RT-qPCR analysis. (**a**) Electropherograms of the PCR products (1—DNA ladder of 250–3000 bp; 2–4—*alkB* amplicons of the expected size of 1219 bp; 5—negative control with water as the template; 6—negative control with the reverse transcription step omitted). (**b**) The relative *alkB* expression ratio compared to *R. ruber* IEGM 231 cells grown in the presence of 1 g/L D-glucose (means ± standard deviations for three biological replicates are shown, and an asterisk (*) shows that the difference in fold change between the two growth conditions is statistically significant at *p* < 0.05). RNA for reverse transcription was obtained after cell lysis using lysozyme with SDS and RNA Solo Kit. Obtained RNA was treated with DNase before cDNA synthesis and PCR.

As can be seen in Figure 7b, *alkB* expression increased 239-fold (log_2_Fold Change = 8) in *R. ruber* IEGM 231 cells degrading *n*-hexadecane, compared to cells grown in the presence of D-glucose. The absence of *alkB* amplicons in RNA samples used directly for PCR with *alkB* primers, without reverse transcription (Figure 7a), was evidence that the DNA was properly removed. The abundance of *alkB* amplicons was related to the number of *alkB* gene transcripts in the sample. These results confirmed the involvement of alkane-1-monooxygenases AlkB in the oxidation of *n*-hexadecane by rhodococci and demonstrated the high quality and feasibility of total RNA for downstream gene expression analysis.

## 3. Discussion

The results obtained allow the working hypothesis to be evaluated. Based on the pronounced hydrophobicity, high lipid content, and lipophilic cell wall of *Rhodococcus* and *Gordonia* bacteria (Figure 1, Table 1), the preliminary cell lysis step was indispensable, and direct exposure to chaotropic salts alone was insufficient to lyse actinomycete cells and extract RNA from them. Chaotropic agents, principally guanidinium thiocyanate, are the active lysis component of most RNA extraction protocols. They disrupt hydrogen-bonding networks and denature proteins, acting as lysis agents; at the same time, they immediately inactivate nucleases, thereby protecting RNA once it is released [18,19,20]. Electron microscopy studies demonstrated that while such agents readily disrupt cytoplasmic membranes, they leave the rigid walls of Gram-positive bacteria, particularly *B. subtilis* and *Micrococcus luteus*, structurally intact even at 4 M concentration [19]. In *Rhodococcus* and *Gordonia* cells, this limitation is compounded by a specific cell envelope structure and composition. A peptidoglycan layer covalently linked to arabinogalactan, itself esterified with long-chain (C_30_–C_60_) mycolic acids, is overlaid by lipid- and polysaccharide-rich EPS around the cells [15,17,57,58,59]. This thick hydrophobic cell wall saturated with lipids and free fatty acids could protect cells from disruption during thawing-freezing cycles, prevent disintegration of cells in a homogenizer, and hinder contact between the cell surface and lysis buffer components (such as guanidinium thiocyanate or lysozyme), as well as preventing their penetration through the cell wall. The intrinsic hydrophobic nature of *Rhodococcus* cells could explain why lysis failed using standard protocols for commercial RNA isolation kits and freezing-thawing cycles. The freezing-thawing released no detectable nucleic acids from any strain, consistent with a peptidoglycan-arabinogalactan-mycolate complex that is mechanically resistant to ice-crystal damage.

The hypothesis that mechanical force would disrupt the envelope of *Rhodococcus* and *Gordonia* cells and facilitate RNA release was not actually confirmed under our experimental conditions. Bead beating did release nucleic acids, and the rates of successful RNA isolation were achieved at 81% in the case of the RNA Solo Kit, but RNA integrity was significantly compromised, and the success of isolating integral RNA was inconsistent (7–17%, see Table 2).

To understand why bead beating performed worse here than in several previously published protocols (see examples in Table S1), we estimated the mechanical energy delivered under the different regimes tested. Approximating each bead as a sphere striking the sample once per oscillation cycle, the kinetic energy E (J) per collision was:

$$E=\frac{1}{2}mv^{2}=\frac{1}{2}\left(\rho V\right)v^{2}=\frac{1}{2}\left(\rho \cdot \frac{4}{3}\pi r^{3}\right)v^{2},$$

where m—mass of a bead (kg); v—velocity (m/s); ρ—density of stainless steel (7.8 × 10^3^ kg/m^3^); V—volume of a bead (m^3^); and r—radius of a bead (m). Therefore, E scales with the cube of bead radius but only with the square of velocity. For our standard 1 mm beads (mass ≈ 4.1 mg) at 5 m/s, this amounts to ≈5 × 10^−5^ J per bead per impact (total 5 × 10^−4^–2 × 10^−3^ J for 10–40 beads). Increasing the bead diameter to 3 mm, while keeping speed constant, raises the mass ~27-fold and the energy per impact to ≈1.4 × 10^−3^ J (total 0.014 J for 10 beads)—almost two orders of magnitude more than the corresponding change in speed (5 to 6 m/s). Assuming an oscillation frequency of 20 Hz and integrating over the tested exposure times, the cumulative mechanical energy delivered to a sample ranged from ≈1.2 J for the standard bead beating protocol to ≈23.8 and ≈24.5 J for the regimes using 3 mm beads or 40 beads, respectively. This is many orders of magnitude above the energy of a single covalent bond (~10^−19^ J) and more than sufficient to fragment nucleic acids once exposed. The calculations are approximate, since the true collision frequency and the fraction of bead energy actually transferred to cells rather than dissipated in the liquid are not known precisely.

Critically, this excess mechanical energy appears to explain not only the poor RNA integrity itself, but also why chemical protection could not rescue it. We attempted to stabilize the released RNA by resuspending cells in 4 M guanidinium thiocyanate; however, this modification did not improve RNA integrity (Figure 2). Because fragmentation under these regimes was mechanical rather than enzymatic—RNA molecules are physically sheared by hydrodynamic forces and impacts that lyse the cells—no chaotropic or other enzyme-inhibiting additives can be expected to prevent it. Consistent with this, pH adjustment of the homogenate was similarly ineffective. It provided conditions for RNA stabilization but did not result in an increase in the successful RNA isolation rates (Figure 2).

This apparent excess of mechanical force relative to what actinomycete cells actually require for lysis is not unique to our study. For example, one study [30] used 10 g of 0.2 mm glass beads with a single 15 mm zirconia-silica ball to recover integral RNA from the soil strain *R. jostii* RHA1. A kinetic energy delivered by one glass bead is ≈8.4 × 10^−8^ J (total 0.08 J for 10 g) and by a zirconia-silica ball is ≈0.05 J. Therefore, cumulative energy appears to be similar or even superior to our study. In *N. europaea*, aggressive sonication (pulses of 3 × 20 s at 40% amplitude) produced a fivefold higher RNA yield than the manufacturer’s protocol but only poor integrity (RNA Integrity Number (RIN) 2–5), while a gentler regime (35% amplitude) improved integrity (RIN 7.7) at the cost of a yield low enough to be barely usable (<10 ng/µL) [21]. Similarly, glass-bead disruption of *S. agalactiae* in a RETSCH MM30 mill (30 Hz, 20 min) yielded only moderate integrity (RIN 5.5) [29], and reducing bead diameter to 0.106 mm in the same study did not reliably improve on this result—indicating that switching to smaller, non-metallic beads for the kinetic energy decrease is not by itself a guarantee of success. Some studies have proposed metal magnetic beads as an alternative for RNA extraction [60], which combine metal density with a more controlled, lower-shear mode of agitation. Moreover, no details on rates of RNA isolation are present in the published examples, and reliable comparison of mechanical energies produced during mechanical disintegration is practically effortful.

It is worth stating here that the densitometric 23S:16S rRNA ratio used throughout this study for RNA integrity assessments is a relative index of rRNA band intensity, not equivalent to RIN. Electrophoresis itself can introduce further degradation not captured by subsequent image analysis, and a ratio below the conventional benchmark of ~1.5 does not necessarily indicate degradation. It is known for several bacteria that 23S rRNA can be natively fragmented in their cells, lowering the apparent ratio even in fully intact RNA [56].

Enzymatic lysis with lysozyme was an alternative approach that proved less damaging to RNA than bead beating. Lysozyme was used at a concentration of 40 mg/mL and for a duration of 1 h, exceeding those previously reported for *Rhodococcus* or other bacteria, where 0.1–20 mg/mL for 10–30 min is typical [21,23,24,39,60]. In the study [39], a lysozyme concentration of 2 mg/mL and incubation for 10–15 min at room temperature failed to disrupt *R. erythropolis* cells. We made an order-of-magnitude estimate of the dynamics of the lysozyme reaction and diffusion comparing this and our regimes.

At 40 mg/mL, the 100 µL reaction volume contains roughly 1.7 × 10^17^ lysozyme molecules (molecular mass 14.3 kDa). Single-molecule studies of lysozyme acting on peptidoglycan report productive turnover rates of 20–50 s^−1^, with non-productive binding events occupying up to 43% of the enzyme’s time on cross-linked substrate [61]. Because the native bacterial cell wall is a considerably poorer substrate than the purified peptidoglycan, we roughly approximated effective turnover rates ≈ 1 s^−1^ to account for reduced substrate accessibility. Even with this conservative value, the enzyme pool has the theoretical capacity to cleave on the order of 10^20^ glycosidic bonds over 1 h. The linear sizes of averagely rod-shaped *Rhodococcus* cells were 0.5–0.6 µm wide and 2–12 µm long [3], giving a surface area of ≈3.1 × 10^6^ to 2.3 × 10^7^ nm^2^ per cell. Peptidoglycan disaccharide (glycosidic β-1,4-linked residues of N-acetylmuramic acid and N-acetylglucosamine) units occupy an axial length of ≈1 nm along a glycan strand [62,63], with adjacent strands spaced ≈2–3 nm apart [63], giving an area of ≈2–3 nm^2^ per disaccharide unit and ≈1.0 × 10^6^ to 1.1 × 10^7^ disaccharide units per single glycan layer. A single-layer thickness of ≈2–4 nm [63,64] and a ~50 nm-thick [65] peptidoglycan carcass of *Rhodococcus* comprising ≈13–25 stacked layers give ≈1.3 × 10^7^ to 2.8 × 10^8^ disaccharide units, or ≈1.3 × 10^7^ to 5.7 × 10^8^ glycosidic bonds per cell. Across the ~10^8^–10^9^ cells present in a 10 mg biomass sample, this corresponds to a total of roughly 1.3 × 10^15^ to 5.7 × 10^17^ glycosidic bonds requiring hydrolysis—several orders of magnitude below the theoretical catalytic capacity of the lysozyme pool.

The lower lysozyme concentration of 2 mg/mL for 15 min would in principle retain a large excess of catalytic capacity over what is needed, since the difference with our regime was just 20-fold for the concentration and 4-fold for the time. The temperature, which was different in the two regimes, appeared not to be critical for the lysis failure of *R. erythropolis*. Lysozyme retains most of its lytic activity at room temperature used in [39] relative to 37 °C used in our study, showing a ~10–15% decrease in activity at room temperature (25 °C) versus 37 °C [66]. The most probable barrier, which may hinder lysozyme hydrolysis of peptidoglycan in *Rhodococcus* and *Gordonia* cells, is assumed to be the EPS produced by these bacteria. The thickness of *Rhodococcus* EPS is declared to be 340–3500 nm, including tightly bound and more diffuse loosely bound EPS [65]. The diffusion did not appear to prevent lysozyme action. Using the Stokes–Einstein relation and the size of lysozyme (Stokes radius ≈ 1.9 nm), free diffusion of this enzyme in water would take milliseconds to cross this distance and reach peptidoglycan. Diffusion within the dense EPS matrix may be retarded by one to two orders of magnitude, but the predicted crossing time still remains on the order of a second, not an hour. Unlike diffusion, electrostatic sequestration of lysozyme by EPS is a plausible explanation. Lysozyme is strongly cationic (net charge ≈+8 at neutral pH), while rhodococcal EPS are typically anionic, with uronic and pyruvic acid content reported up to 18% and 16% of the EPS mass [65]. A rough charge-balance estimate, based on the tightly bound EPS shell thickness and uronic + pyruvic acid content, suggests the available carboxylate groups could electrostatically trap on the order of 10^12^–10^16^ lysozyme molecules—up to 27% of the enzyme pool at 40 mg/mL and up to 100% at 2 mg/mL of lysozyme. This mechanism is documented for human lysozyme, where anionic biopolymers sequester and inactivate the protein through nonspecific electrostatic complexation [67].

This interpretation is consistent with reports that *Rhodococcus equi* cell walls display measurable resistance to lysozyme lysis [68], and it offers one plausible, quantitatively grounded explanation for why the brief, low-concentration lysozyme protocols established for other Gram-positive bacteria [23,24,25,27,60] may have failed for *Rhodococcus* [39]. This also points to why the present protocol succeeds: rather than applying a brief exposure at a concentration calibrated for other Gram-positive bacteria, the extended, high-concentration regime used here appears sufficient to saturate the EPS electrostatic sequestration capacity while still leaving enough active enzyme to reach and hydrolyze the underlying peptidoglycan. Once access was achieved, lysozyme treatment itself did not further damage RNA: electropherograms of lysozyme-lysed samples lacked the smearing characteristic of mechanical shearing, consistent with RNA remaining shielded within an at least transiently intact cytoplasmic membrane until it is exposed to the extraction buffer.

In studies [23,24], 1.2% Triton X-100 or SDS was used to lyse *E. faecalis*, *M. smegmatis*, *S. aureus*, *S. epidermidis* cells, and *B. subtilis* spores alongside the lysozyme. The hypothesis that detergent in a high concentration would be required to solubilize the lipid-rich cell wall was not confirmed as a general rule in this study. RNA isolation success and integrity did not differ significantly between cell lysis with and without SDS. This may indicate that, once lysozyme has sufficiently degraded the peptidoglycan, the cytoplasmic membrane is already adequately solubilized by the kit’s lysis buffer. It makes an additional anionic detergent largely redundant for most strains and growth conditions, including growth on long-chain *n*-alkanes, NSAIDs, or organic solvents, all of which increase cellular lipid content (Figure 1).

The critical condition was only growth of *Rhodococcus* cells on used frying oil. The cells formed aggregates, which were difficult to harvest, apparently due to intense emulsification of oil triacylglycerols and their derivatives by cell-bound trehalose lipid biosurfactants produced by these bacteria in the presence of liquid hydrophobic substrates [69]. To overcome this effect, a detergent was essential. Tween 80 in a low concentration (0.1%) enabled disintegration and dispersion of cell aggregates for the following cell lysis and RNA isolation. Nonionic Tween 80 in a very low concentration, not anionic SDS, was used for pre-washing to avoid any effects on proteins or the cytoplasmic membrane.

Not only the cell lysis procedure, but also the RNA extraction kit plays an important role in successful RNA isolation from *Rhodococcus* and *Gordonia* cells. TRIzol™ LS was expected to give an advantage over silica column-based kits in dissolving the lipid-rich cell wall that was not approved. At bead beating, RNA isolation success with TRIzol™ LS did not significantly differ from the RNA Solo Kit, but rates of RNA isolation for both kits were 3–5-fold higher than those for the RNeasy Mini Kit (Table 2). However, the integrity of isolated RNA was low for all three kits, indicating that once cells were disrupted by beads, none of the tested extraction chemistries could reliably maintain RNA integrity. The failure of TRIzol™ LS to recover RNA from lysozyme-lysed cells, in contrast to the marked success of the silica column-based RNA Solo Kit, was surprising. This may be related to the different physical principles underlying the two chemistries. TRIzol relies on clean partitioning of RNA into an aqueous phase, away from proteins, DNA, and lipids retained in the organic and interphase layers; incompletely solubilized cell wall fragments, mycolic acid-rich debris, or exopolysaccharide material remaining after lysozyme treatment could aggregate at the interphase and trap RNA there, and phenol-chloroform-based extraction is known to be sensitive to exactly this kind of contamination [20]. Silica column-based kits do not depend on phase separation: RNA is instead selectively adsorbed to a silica matrix under high-salt, ethanol-containing conditions after insoluble debris has been removed by centrifugation, a chemistry inherently more tolerant of residual lipophilic or polysaccharide contamination [20,70].

The comparison of growth conditions offers some mechanistic insight into RNA behavior beyond the extraction protocol itself. HgCl_2_ exposure was associated with a modest but significant reduction in the 23S:16S rRNA ratio relative to *n*-alkane-grown cultures. Mercury(II) is a documented catalyst of RNA strand cleavage and isomerization and can generate reactive oxygen species that produce RNA derivatives and direct strand breaks [35,36,38]. By contrast, cultures grown on *n*-alkanes, which showed slightly higher and more consistent 23S:16S rRNA ratios, were also associated with elevated RNA concentrations in this study, suggesting active, unstressed biosynthesis rather than a specific protective effect of the substrate itself. A similar effect was detected for propane-grown cells. The growth on propane measurably improved bead-beating success, yielding RNA in 59% of trials compared with 7% for all other conditions combined. It is known that formation of an even thicker cell wall and the invaginated cytoplasmic membrane saturated with numerous multi-subunit propane monooxygenase complexes are necessary for assimilation of propane [3]. This finding was in agreement with RNA yields. An increase of RNA yields of 29% was detected in *Rhodococcus* cells grown in the presence of *n*-alkanes compared to those grown on glucose. We hypothesize that assimilation of alkanes involves a correspondingly larger complement of ribosomes or intense transcription of particular mRNA required for synthesis of oxidoreductases and enzymes participating in biosynthesis of mycolic acids and biosurfactants, as well as in metabolism of fatty acids [3,16,69,71]. The resulting higher cellular content of rRNA and mRNA plausibly raised the amount of nucleic acid released and detected.

In conclusion, the specific chemical composition of *Rhodococcus* and *Gordonia* cell walls and the EPS layers defines the success of cell lysis and RNA stability using a high concentration of lysozyme at a long exposure and failure of metal bead homogenization. Practical recommendations may be given, based on the obtained results. The 40 mg/mL of lysozyme for 1 h combined with silica column-based extraction reliably results in isolation of relatively integral RNA. The principal advantage of this protocol over previously published methods for these genera is that its reproducibility is quantified rather than assumed: integral RNA was recovered in 79–97% of trials across 21 strains and 34 growth conditions (Table 2), whereas prior reports for *Rhodococcus* and *Gordonia* typically describe a successful outcome without reporting success or failure rates. The yield, purity, and integrity of the obtained RNA are appropriate for downstream analysis as confirmed in analysis of the *alkB* gene expression (Figure 7). The protocol does have practical limitations, however. The extended incubation increases processing time relative to standard kit protocols, and re-optimization may be required for strongly emulsifying pollutants such as used frying oil. The approach has so far been validated only in *Rhodococcus* and *Gordonia*; whether it extends without modification to other mycolic acid-containing actinomycetes, such as *Mycobacterium* or *Nocardia*, remains to be tested. It is worth noting that contamination of resulting RNA with genomic DNA can be high (Table S4); although commercial kits declare proper component separation. Our study just confirms the necessity to use DNases, which is obligatory for transcriptomics and single-gene expression analysis anyway. Beyond the single-gene validation shown here, the reproducibility and RNA quality achieved make this protocol suitable for RNA-Seq-based transcriptomic studies of *Rhodococcus* and *Gordonia* responses to hydrophobic and toxic growth substrates, including comparative expression analyses across strains and species relevant to biodegradation, biocatalysis and bioremediation.

## 4. Materials and Methods

### 4.1. Chemicals

Salts, solvents, hydrocarbons, urea, LB, LB agar, bacteriological agar for solid mineral media, yeast extract, D-glucose, glycerol, ammonium acetate, hydrogen peroxide and halloysite nanotubes (Al_2_Si_2_O_5_(OH)_4_, outer diameter ~50 nm, inner diameter ~15 nm, and length of 700 nm to 2 μm) had purity ≥ 97% and were purchased from Reachim (Perm, Russia), Diaem (Moscow, Russia) or Sigma-Aldrich (Burlington, MA, USA). Propane (freon r290) was ≥99.5% pure and purchased from NPP Sintez, LLC (Perm, Russia). Used frying oil was obtained from a local KFC fast food restaurant (Perm, Russia). Oleanolic and glycyrrhetinic acids had purity ≥ 98% and were purchased from Thermo Fisher Scientific (Waltham, MA, USA) and TCI (Antwerpen, Belgium), respectively. (–)-Isopulegol was chemically synthesized by the Chemistry Faculty of the Novosibirsk State University (Novosibirsk, Russia). NSAIDs, such as diclofenac, ibuprofen, ketoprofen, meloxicam and naproxen, were used as ultrapure (≥99.99%) drug substances and were purchased from BLD Pharmatech Ltd. (Shanghai, China).

SDS, dimethyl sulfoxide (DMSO), guanidinium thiocyanate, sodium hydroxide, Tween 80, agarose, TAE buffer and GelRed were of molecular biology grade and purchased from AppliChem GmbH (Darmstadt, Germany), Diaem (Moscow, Russia) or Evrogen (Moscow, Russia). Lysozyme was purchased from Helicon (Moscow, Russia). TRIzol™ LS Reagent, RNeasy Mini Kit and RNA Solo Kit were purchased from Invitrogen (Carlsbad, CA, USA), Qiagen (Hilden, Germany) and Evrogen (Moscow, Russia), respectively. The RNA Solo Kit contained integrated DNase E. Additionally, the DNA-free™ DNA Removal Kit was used, and it was purchased from Thermo Fisher Scientific (Waltham, MA, USA). RNA loading buffer was purchased from Servicebio (Wuhan, China). DNA ladders 250–3000 bp and 250–10,000 bp were purchased from Diaem (Moscow, Russia), and RNA ladder 50–1000 nt was purchased from New England Biolabs (Ipswich, MA, USA). Qubit™ RNA BR Assay Kit was purchased from Thermo Fisher Scientific (Waltham, MA, USA).

HiScript^®^ III 1st Strand cDNA Synthesis Kit (+gDNA wiper) for RT-qPCR was purchased from Vazyme (Nanjing, China). 5× qPCRmix HS SYBR was purchased from Evrogen (Moscow, Russia). Primers for RT-qPCR were synthesized at Evrogen (Moscow, Russia).

Sterile RNase-free MilliQ^®^ water (Evrogen, Moscow, Russia) was used for preparation of stock solutions and in all molecular biology steps. Laboratory equipment and working surfaces were preliminary cleaned with RNaseZap™ (Thermo Fisher Scientific, Waltham, MA, USA).

### 4.2. Bacterial Strains and Growth Conditions

A total of 21 strains, such as *G*. *alkanivorans* IEGM 1277, *G*. *paraffinivorans* IEGM 735, *G*. *rubripertincta* IEGM 133, R. cerastii IEGM 1243, *R*. *pyridinivorans* IEGM 639, *R*. *qingshengii* IEGM 267, R. rhodochrous IEGM 757, IEGM 1162, IEGM 1360, IEGM 1362, R. ruber IEGM 231, IEGM 346, IEGM 439, IEGM 560, IEGM 1122, IEGM 1156, *Rhodococcus* sp. IEGM 1351, IEGM 1354, IEGM 1372, IEGM 1379, IEGM 1381 and IEGM 1404, from the Regional Specialised Collection of Alkanotrophic Microorganisms (acronym IEGM, WDCM #768, http://www.iegmcol.ru/, accessed on 29 July 2026) were used in this study. Isolation sources, biotechnological properties and DDBJ/EMBL/GenBank accession numbers of draft genome sequences for these strains are shown in Table S2.

Bacteria were grown in liquid and on solid media at 28 °C and 160 rpm or without shaking for 1–14 days. Liquid media included LB—standard 1× LB and twice concentrated 2× LB, “Kievskaya” (K) and RS minimal salt media. The composition of K (g/L): KH_2_PO_4_—1.0, K_2_HPO_4_—1.0, NaCl—1.0, KNO_3_—1.0, MgSO_4_·7H_2_O—0.2, CaCl_2_—0.02, FeCl_3_·7H_2_O—0.001 (http://www.iegmcol.ru/medium/med08.html, accessed on 29 July 2026). The composition of RS (g/L): K_2_HPO_4_—2.0, KH_2_PO_4_—2.0, KNO_3_—1.0, (NH_4_)_2_SO_4_—2.0, NaCl—1.0, MgSO_4_—0.2, CaCl_2_—0.02, FeCl_3_·7H_2_O—0.001 (http://www.iegmcol.ru/medium/med11.html, accessed on 29 July 2026). Yeast extract in the concentration of 0.1 g/L and the trace element solution (FeCl_3_·7H_2_O—1.5 g/L, H_3_BO_3_—0.1 g/L, ZnSO_4_·7H_2_O—0.01 g/L, Co(NO_3_)_2_·6H_2_O—0.05 g/L, CuSO_4_·5H_2_O—0.005 g/L, and MnCl_2_·4H_2_O—0.005 g/L) in the concentration of 0.1 vol.% were added into mineral salt media. Solid media were LB agar and agarized K medium. The latter was not supplemented with yeast extract and the trace element solution to simulate oligotrophic growth conditions.

Growth substrates, co-metabolic substrates and heavy metal salts were used as follows: gaseous alkanes (propane and *n*-butane) were used as a gas mixture with air in the ratio 1:4 in closed desiccators; liquid alkanes (*n*-pentane, *n*-hexane, *n*-heptane, *n*-octane, *n*-nonane, *n*-decane, *n*-undecane, *n*-dodecane, *n*-tridecane, *n*-tetradecane, *n*-pentadecane, *n*-hexadecane and pristane), monoaromatic hydrocarbons (benzene and *o*-xylene), (–)-isopulegol, ketoprofen, glycerol and used frying oil were added in concentrations of 0.001–0.1 vol.%; carbohydrates (D-glucose), an organic acid salt (ammonium acetate), polyaromatic hydrocarbons (naphthalene and phenanthrene), oleanolic acid, glycyrrhetinic acid, meloxicam, naproxen, and a NSAID mixture were added in concentrations of 0.01–20.0 g/L; and HgCl_2_ was added in the concentration of 10.9 or 86.9 mg/L. Terpenoids (oleanolic acid, glycyrrhetinic acid and (–)-isopulegol) were preliminarily dissolved in DMSO at a concentration of 100 mg/mL. The NSAID mixture contained diclofenac, ibuprofen and naproxen in equal amounts. NSAIDs were preliminarily dissolved in ethanol at a concentration of 20 mg/mL. Complete details of growth conditions are provided in Table S3.

In particular experiments on biotransformation of terpenoids, resting cells and halloysite nanotubes were used [10]. To obtain resting cells, 1–5 mL of a bacterial culture at the early stationary growth phase was centrifuged at 12,000× *g* for 4 min, washed in 500 µL of 10% SDS or in 500 µL of 0.5% NaCl, and then resuspended in 100 µL of water, 100 µL of 4 M guanidinium thiocyanate, or the pellet was used. A suspension of halloysite nanotubes at their final concentration of 0.1 g/L was used to enhance the biotransformation process of (–)-isopulegol [10].

### 4.3. Determination of Physical-Chemical Characteristics of Cells and Total Cellular Lipids

In all analyses, bacteria were preliminarily washed twice with 0.5% NaCl. Hydrophobicity of cells was determined using MATH test and SAT in accordance with recommendations published elsewhere [72,73,74].

For MATH-test, cells were resuspended in PUM buffer (K_2_HPO_4_·3H_2_0—2.20 g/L, KH_2_PO_4_—7.26 g/L, urea—1.80 g/L, MgSO_4_·7H_2_0—0.20 g/L, pH 7.1) to the optical density A_600 nm_ = 0.5. A cell suspension was mixed with *n*-hexadecane by vortexing (Vortex FS 16, BioSan, Riga, Latvia) for 2 min. The volume ratio *n*-hexadecane:a cell suspension was 1:2.5. The mixture was settled for 24 h, and the optical density of the aqueous phase was measured at 600 nm. The cell suspensions without *n*-hexadecane and the sterile buffer with *n*-hexadecane were used as controls. The degree of hydrophobicity was defined as a percentage of cells adhering to the hydrocarbon phase.

For SAT, the 10 μL bacterial suspension in MilliQ^®^ water (A_600nm_ ~0.5–1.0) was gently mixed with 10 μL of 0.2–2.0 M (NH_4_)_2_SO_4_ on a microscope slide. Cell aggregation was recorded by phase contrast microscopy (Axiostar Plus, Zeiss, Jena, Germany) using a 100× immersion lens (numerical aperture 1.4). A minimum of 3 fields of each variant were examined. The method uses the principle that the more hydrophobic the bacterial cell wall, the lower the salt concentration at which cell aggregation was observed. To determine a level of hydrophobicity, a scale was used: high hydrophobicity—the salinity index of an ammonium sulfate solution ranging from 0 to 0.8 M; moderate—from 1.0 to 2.0 M; weak—>2.0 M.

Total cellular lipids were determined gravimetrically after chloroform-methanol extraction from dry biomass as described [75]. The 50 mg of cell biomass was suspended in 1 mL distilled water. The chloroform-methanol mixture (4 mL) was added to the suspension (1:2), shaken for 2 min in a Vortex FS 16 shaker at 2400 rpm, and left to stand for 24 h. Then the mixture was centrifuged at 3000 *g* for 10 min and the precipitate was extracted again with 5 mL of the chloroform-methanol-water mixture (1:2:0.8). The supernatants were combined and centrifuged upon the addition of 5 mL of chloroform-water mixture (1:1). The chloroform layer of each sample was quantitatively transferred to weighted round bottomed 50 mL flasks, diluted with an equivalent amount of benzene, and evaporated in a rotor evaporator (Heidolph, Schwabach, Germany) at 60 °C. The flask was weighed after reaching a constant mass on an AUW 120D high-precision analytical balance (Shimadzu, Kyoto, Japan).

The zeta potential of cells was estimated by the dynamic light scattering technique using a ZetaSizer Nano ZS analyzer (Malvern Instruments, Malvern, UK) and the Malvern ZetaSizer software, v. 2.2 (Malvern Instruments, Malvern, UK). Washed cells were resuspended in 0.1 M KNO_3_ (pH 7.0) until OD_600nm_ was 0.2. Measurements were carried out in a U-shaped cuvette with gold-plated electrodes at 25 °C. Zeta-potential was calculated using the following equation:

ζ = (ηu)/(ε_r_ε_0_)

where ζ—zeta-potential, V; u—electrophoretic mobility, m^2^/(V·s); η—viscosity, N·s/m^2^; ε_0_—dielectric permittivity in vacuum, F/m; ε_r_—relative dielectric permittivity.

### 4.4. Extraction of Total RNA

The 2–4 mL of bacterial cultures or bacterial cell suspensions were centrifuged at 6000 rpm for 15 min; the liquid was thoroughly removed, and cell pellets were further used. Optionally, cells were pre-washed with 500 µL of 0.1 vol.% Tween 80 and subsequently with 500 µL of 0.5% NaCl. Then, cells were directly subjected to RNA extraction procedures or preliminarily lysed. The initial amount of biomass used for RNA extraction was 10 ± 1 mg of wet cell weight.

Cell lysis protocols are described below.

(1) Freezing-thawing lysis. Cells were resuspended in 100 µL deionized water and subjected to three freezing-thawing cycles. One cycle included incubation at −20 °C for 20 min and then at 96 °C for 20 min.
(2) Metal bead lysis. Cells were resuspended in 100 µL deionized water or 4 M guanidinium thiocyanate. Then, cells were mechanically disrupted using 10, 15, or 40 stainless steel beads with a diameter of 1 or 3 mm (Changzhou Feige Steel Ball Co., Ltd., Changzhou, China) in a FastPrep-24 5G Homogenizer (MP Biomedicals, Santa Ana, CA, USA) at 5 or 6 m/s for 1, 2, or 10 min. Metal beads were preliminary washed once with 35% hydrogen peroxide and then three times with MilliQ^®^ water. New beads were used for each extraction. Metal beads are heavy and deliver a high mechanical energy. The number of beads was selected sterically to provide intense movement in 100 μL during homogenization.
(3) Lysozyme lysis. Cells were resuspended in 100 µL of a 40 mg/mL lysozyme solution and incubated at 37 °C for 1 h. The 10% SDS was added optionally in 10 µL aliquots until a clear solution was obtained. Lysozyme and SDS concentrations, as well as incubation time, were selected to exceed those in the literature [21,23,24,39,60].

RNA isolation kits (TRIzol™ LS Reagent, RNeasy Mini Kit and RNA Solo Kit) were used for total RNA extraction from native cells or cell lysates according to manufacturers’ protocols. TRIzol™ LS Reagent was a convenient TRIzol reagent, being a monophasic solution of phenol and guanidinium thiocyanate and required chloroform for RNA separation. Occasionally, the pH of lysates was checked with pH paper (Lachner, Ltd., Neratovice, Czech Republic) and adjusted using 100 µL of 1 M NaHCO_3_ or 100 µL of 1 M NaOH prior to phase separation, followed by TRIzol protocols. RNeasy Mini Kit and RNA Solo Kit were silica column-based reagents. DNA was removed from RNA preparations via DNase digestion performed with the DNA-free™ DNA Removal Kit or DNase E from RNA Solo Kit following manufacturer’s instructions.

Preliminary integrity of RNA was estimated using electrophoresis in the 1.2% agarose gel. Prior to loading, a 5 µL RNA sample was mixed with 5 µL 2× RNA loading buffer, incubated at 70 °C for 1 min, then placed on ice, and 10 µL was loaded into the gel according to the protocol (https://evrogen.ru/support/technologies/rna-electrophoresis, accessed 29 on July 2026). Samples were run at 70 V for 40 min. GelRed was used for visualization of nucleic acid bands. RNA integrity index was calculated as the 23S:16S rRNA ratio [56] using a densitometric analysis [76]. Gel images were processed in Python 3.12 using OpenCV 4.13 and SciPy 1.17. Each lane was defined as an equal-width vertical strip of the gel image; a mean pixel-intensity profile was calculated along the electrophoretic migration axis for each lane. Profiles were smoothed with a Gaussian filter (σ = 4 px) and background-corrected using a rolling 20th-percentile local baseline. The two most prominent local intensity maxima within each lane, corresponding to the 23S and 16S rRNA bands, were identified by peak-prominence analysis; band area was calculated by trapezoidal integration of the baseline-subtracted signal within a fixed ±18-pixel window around each peak. The 23S:16S ratio was calculated as the area ratio of the upper (23S) to lower (16S) peak. This pixel-densitometry approach is analogous in principle to gel-image quantification performed in [76]. RNA purity (A260/A280 and A260/A230 ratios) was evaluated using a NanoPhotometer N50 (Implen, München, Germany). RNA concentrations were determined with a Qubit 4 fluorometer (Thermo Fisher Scientific, Waltham, MA, USA) after sample preparation with the Qubit™ RNA BR Assay Kit. The volume of eluate with total RNA for all analyses was 50 μL.

### 4.5. Quantitative Real-Time PCR Analysis

Total RNA was extracted from *R. ruber* IEGM 231 cells grown in RS with 1 g/L D-glucose or 0.1 vol.% *n*-hexadecane for 48 h. For both growth conditions, cells were in the late exponential phase, which was determined by growth curves. Genomic DNA was removed from total RNA using DNase E from the RNA Solo Kit. The purity of RNA was appropriate: A260/280 > 1.8 and A260/230 ≥ 2.0. cDNA was then synthesized using HiScript^®^ III 1st Strand cDNA Synthesis Kit according to the manufacturer’s protocol (Table S5). The genomic DNA removal step included in the kit was omitted, as this nucleic acid had already been eliminated during the RNA extraction procedure.

The RT-qPCR was performed for the target gene *alkB*, coding for alkane-1-monooxygenase, and the reference genes *gyrB*, coding for the subunit B of DNA-gyrase, and 16S rRNA gene using a CFX Connect Real-Time PCR Detection System (Bio-Rad, Hercules, CA, USA). Primer sequences are shown in Table 4. Primers for *alkB* and *gyrB* genes were constructed using Primer–BLAST (NCBI, Bethesda, MD, USA). The primers 27F and 1492R were used to amplify the 16S rRNA gene [77]. The PCR master mix was prepared using 5× qPCRmix HS SYBR according to the manufacturer’s instructions. The composition of the final reaction mixture was following: 5× qPCRmix-HS SYBR—2 μL, forward primer (FP, 10 μM)—0.5 μL, reverse primer (RP, 10 μM)—0.5 μL, cDNA (1 μg/μL)—2 μL, and nuclease-free water—5 μL. The final volume of the PCR mixture was 10 µL. The final initial concentration of cDNA in each PCR was 0.2 μg/μL. The RT-qPCR was performed by following steps: 95 °C for 3 min, followed by 34 cycles of DNA denaturation at 95 °C for 30 s, annealing at 60–70 °C (gradient) for 30 s, extension at 72 °C for 30 s, and a final extension at 72 °C for 10 min.

The 16S rRNA gene and *gyrB* were selected from a total of four genes—16S rRNA gene, *gyrA*, *gyrB,* and *dnaG*. Primers for all genes and values of the PCR efficacy are shown in Table S6. Distribution of C_t_ values for the candidate reference genes is shown in Figure S1. Data on expression stability of the candidate reference genes for normalization of RT-qPCR results are presented in Figure S2; and electropherograms of the reference gene PCR products are shown in Figure S3. The 16S rRNA gene and *gyrB* were selected as the reference genes on the basis of their stability and the C_t_ values (stability of *dnaG* was better than the 16S rRNA gene, but C_t_ was close to the detection level, see Figures S1 and S2).

Relative expression levels of the target gene *alkB* were calculated using the efficiency-corrected relative quantification method described by Pfaffl [78] (Table S7). Melting curves for *alkB*, *gyrB,* and 16S rRNA gene are shown in Figure S4. Two types of negative controls were included in each experiment: (1) no template control containing nuclease-free water instead of cDNA to detect contamination of the reaction mixture and (2) no reverse transcriptase control for each RNA sample to exclude the possibility of genomic DNA amplification [79]. Data acquisition and analysis were performed using CFX Manager Software 3.1 (Bio-Rad, Hercules, CA, USA).

### 4.6. Statistics

All experiments (each strain, each growth condition) were performed in 3–12 biological replicates in independent trials, and each trial included three biological replicates. RNA was isolated from each replicate. On agarose gel images (Table S4) each experimental lane mainly corresponded to one biological replicate. In case a sample was run in the gel more than one time, technical replicates were mentioned for these gels (see Table S4). One blank sample (abiotic control without cells) was used in each trial to exclude RNA background signal. No RNA was detected in these samples by electrophoresis and a fluorometric measurement.

The design of the study includes step-by-step-conducted experiments: no cell lysis in combination with TRIzol™ LS Reagent → thawing-freezing cycles combined with TRIzol™ LS Reagent or a silica column-based kit → bead beating combined with TRIzol™ LS Reagent or a silica column-based kit → lysozyme with and without SDS combined with TRIzol™ LS Reagent or a silica column-based kit. If integral RNA (visible 23S and 16S rRNA bands) was not recovered from one of nine samples (three independent trials under different growth conditions for the same strain and three biological replicates in each trial), this combination of lysis method and RNA extraction kit was not used further.

Differences in zeta potential, hydrophobicity levels and total lipids were assessed using a two-tailed Student’s *t*-test. Differences in the probability of successful and integral RNA isolation between lysis methods and extraction kits were assessed using Fisher’s exact test, with Holm correction applied for multiple pairwise comparisons. The effect of growth condition on the probability of RNA isolation was assessed using the χ^2^ test. Differences in the 23S:16S rRNA ratio among growth conditions were assessed using the Kruskal–Wallis test, followed by pairwise Mann–Whitney *U* tests with Bonferroni correction. Associations between RNA integrity, purity and concentration were assessed using Spearman’s rank correlation coefficient. All differences and effects detected were significant at *p* < 0.05. Statistica version 13.5.0.17 (TIBCO Software Inc., Palo Alto, CA, USA), Microsoft^®^ Excel^®^ 2019 (Microsoft Corporation, Redmond, WA, USA), R version 4.6.1 [80] and Claude Sonnet 5 (Anthropic, San Francisco, CA, USA) were used to calculate basic statistics, perform the tests described above, calculate 23S:16S rRNA ratios and visualize data.

## 5. Conclusions

This study identifies the hydrophobic and lipid-rich cell envelope as the primary barrier to RNA extraction from *Rhodococcus* and *Gordonia* actinomycetes, and shows that this barrier is not overcome by mild physical treatment: extraction without prior lysis, freeze–thaw cycling, and metal bead homogenization all failed to reliably yield intact RNA, with bead beating in particular producing excessive, uncontrolled mechanical shear that degraded RNA regardless of chemical protection. In contrast, enzymatic lysis with a high concentration of lysozyme, followed by extraction with a silica column-based kit, consistently yielded RNA of sufficient purity, integrity, and quantity for downstream analysis, as validated directly by RT-qPCR detection of the *alkB* gene induction during *n*-hexadecane assimilation.

Beyond establishing a working protocol, this study offers a mechanistic account of why the tested approaches succeed or fail—grounded in the physical and chemical properties of the actinomycete cell envelope rather than empirical trial and error. This includes a quantitative explanation for bead-beating failure based on excess mechanical energy, and a kinetic and electrostatic analysis indicating that lysozyme’s limited efficacy is more plausibly explained by sequestration within the anionic EPS layer than by insufficient catalytic capacity or restricted diffusion.

The protocol’s reliability was demonstrated across a biotechnologically relevant set of *Rhodococcus* and *Gordonia* strains and growth conditions, including hydrophobic and recalcitrant substrates and heavy metal exposure. Whether this reliability extends to other actinomycete genera or to more extreme growth conditions than those tested here was not directly assessed in this study and remains to be established. Likewise, extending the validated protocol to RNA-Seq applications, more detailed analysis of RNA integrity, testing a hypothesis of lysozyme sequestration within the EPS produced by *Rhodococcus* and *Gordonia* cells, and morphological and dynamics studies of the cell lysis during bead beating and lysozyme exposure are directions for future work.

## Figures and Tables

**Table 1:** Zeta potential of *Rhodococcus* and *Gordonia* cells under different growth conditions.

Strain	Growth Conditions	Zeta Potential, mV
*G. alkanivorans* IEGM 1277	RS + 0.1 vol.% *n*-hexadecane (control)	−6 ± 0
*G. alkanivorans* IEGM 1277	RS + 0.01 g/L meloxicam + 0.1 vol.% *n*-hexadecane	−21 ± 0 *
*R. cerastii* IEGM 1243	LB	−26 ± 1
*R. qingshengii* IEGM 267	LB (control)	−27 ± 2
*R. qingshengii* IEGM 267	RS + 0.1 vol.% *n*-hexadecane	−16 ± 1 *
*R. rhodochrous* IEGM 757	LB	−37 ± 1
*R. rhodochrous* IEGM 1162	LB	−37 ± 1
*R. rhodochrous* IEGM 1362	LB (control)	−37 ± 2
*R. rhodochrous* IEGM 1362	RS + 0.1 g/L of yeast extract	−37 ± 7
*R. rhodochrous* IEGM 1362	RS + 0.025 vol.% (–)-isopulegol + 0.1 g/L yeast extract	−33 ± 8
*R. ruber* IEGM 231	LB	−32 ± 1
*R. ruber* IEGM 346	RS + 5 g/L D-glucose (control)	−35 ± 2
*R. ruber* IEGM 346	RS + 0.01 g/L NSAIDs + 5 g/L D-glucose	−31 ± 1

Means ± standard deviations for three biological replicates are shown. * Difference from the control for this strain is statistically significant at *p* < 0.05 (*t*-test). The NSAID mixture contains diclofenac, ibuprofen, and naproxen (0.01 g/L each). Growth conditions are detailed in Table S3 and the Materials and Methods Section.

**Table 2:** Success rates of RNA isolation depending on the cell lysis method and RNA extraction kit.

Lysis Method	RNA Extraction Kit	Total Number of Samples	Number of Samples with RNA Isolated ^1^	Number of Samples with Integral RNA	Number of Samples Without RNA Isolated ^1^	Probability to Isolate RNA, %	Probability of Integral RNA Isolation, %
Without lysis	TRIzol™ LS Reagent	9	0	0	9	0	0
Without lysis	RNeasy Mini Kit	9	0	0	9	0	0
Freezing-thawing	TRIzol™ LS Reagent	9	0	0	9	0	0
Bead beating	TRIzol™ LS Reagent	21	12	2	9	58 ^3^	17 ^2^
Bead beating	RNeasy Mini Kit	171	29	3	142	17 ^4^	10 ^2^
Bead beating	RNA Solo Kit	18	15	1	3	81	7 ^2^
Lysozyme without SDS	TRIzol™ LS Reagent	9	3	0	6	33 ^5^	0
Lysozyme with SDS	TRIzol™ LS Reagent	9	0	0	9	0	0
Lysozyme without SDS	RNA Solo Kit	36	35	35	1	97	100
Lysozyme with SDS	RNA Solo Kit	66	54	52	12	82	96

Bead beating includes all protocol modifications. TRIzol™ LS and RNA Solo Kit were used only with the standard protocol of bead beating. RNeasy Mini Kit was used with the standard and modified bead beating protocols that explain the highest number of samples for this combination. “Integral RNA” here is RNA with distinct 23S and 16S rRNA bands, although actual integrity can be low or moderate. Each sample corresponds to one biological replicate. Combinations of lysis method and RNA extraction kit, which did not recover integral RNA from 9 samples (three independent trials and three biological replicates in each trial), were not used further. “Probability to isolate RNA” = (number of samples with RNA isolated, of any quality/total number of samples) × 100; “Probability of integral RNA isolation” = (number of samples with integral RNA/number of samples with RNA isolated) × 100. ^1^ The presence and absence of RNA was additionally confirmed using a fluorometric analysis. ^2^ Differ from lysozyme treatment, ^3^ differ from lysozyme treatment and bead beating + RNeasy, ^4^ differ from all other variants, and ^5^ differ from RNA Solo Kit isolations at *p* < 0.05 in pairwise comparisons (Fisher’s exact, Holm-corrected, comparisons between groups with isolated RNA).

**Table 3:** Densitometrically estimated 23S:16S rRNA ratios depending on the cell lysis method and RNA extraction kit.

Lysis Method	RNA Extraction Kit	*n*	Median	Range (Min–Max)
Bead beating	TRIzol™ LS Reagent	2	0.86	0.82–0.90
Bead beating	RNeasy Mini Kit	3	1.90	1.70–2.16
Bead beating	RNA Solo Kit	1	1.26	1.26
Lysozyme without SDS	RNA Solo Kit	35	1.38	0.78–3.97
Lysozyme with SDS	RNA Solo Kit	52	1.53	0.90–2.95

The *n* is the number of samples (biological replicates) analyzed, which contain RNA determined preliminarily as integral (see Table 2).

**Table 4:** Primers used for RT-qPCR.

Gene	Role in PCR	Coded for	PCR-Product Size, bp	Forward Primer (FP) Sequence	Reverse Primer (RP) Sequence	Reference
*alkB*	Target gene	Alkane-1-monooxygenase	1219	5′-GACGACGTCGGACATCG-3′	5′-CTGTGCTCCGTACTGCG-3′	This study
*gyrB*	Reference gene	Subunit B of DNA-gyrase	1494	5′-GTCACTGATGCCACGAAGGAGGTA-3′	5′-TACTAGCGGACCTGGAGCAACAAG-3′	This study
16S rRNA gene	Reference gene	16S rRNA	1465	5′-AGAGTTTGATCCTGGCTCAG-3′	5′-CGGTTACCTTGTTACGACTT-3′	[77]

## Data Availability

All data generated or analyzed during this study are included in this published article and its Supplementary Information files. Other data can be shared on request.

## Supplementary Materials

The following supporting information can be downloaded at: https://www.mdpi.com/article/10.3390/ijms27177890/s1.

## Abbreviations

The following abbreviations are used in this manuscript:

bp	Base pair(s)
cDNA	Complementary DNA
DDBJ/EMBL/GenBank	DNA Data Bank of Japan/European Molecular Biology Laboratory/GenBank (depositories for sequences)
DMSO	Dimethyl sulfoxide
EPS	Extracellular polymeric substances
FP	Forward primer
GT	Guanidinium thiocyanate
IEGM	Institute of Ecology and Genetics of Microorganisms (acronym of the Regional Specialised Collection of Alkanotrophic Microorganisms)
K	“Kievskaya” minimal salt medium
LB	Luria-Bertani (broth)
MATH	Microbial Adhesion to Hydrocarbons
nt	Nucleotide(s)
NSAIDs	Non-steroidal anti-inflammatory drugs
PCR	Polymerase chain reaction
PUM (buffer)	Phosphate–urea–magnesium sulfate buffer
RIN	RNA Integrity Number
RP	Reverse primer
RS	“*Rhodococcus*-surfactant” minimal salt medium
RT-qPCR	Reverse transcription–quantitative polymerase chain reaction
RNA-Seq	RNA sequencing
SAT	Salt Aggregation Test
SDS	Sodium dodecyl sulfate
TAE (buffer)	Tris-acetate-EDTA buffer
WDCM	World Data Centre for Microorganisms
