# Supplementary material for: Cell Disruption and RNA Extraction from Rhodococcus and Gordonia Bacteria Grown in the Presence of Hydrophobic Organic Compounds and Heavy Metal Salts
Source: Int J Mol Sci. 2026 Sep 4;27(17):7890. doi: 10.3390/ijms27177890 (PMC13565763; doi:10.3390/ijms27177890)
Supplement: Supplementary file 1 [file ijms-27-07890-s001.zip › ijms-4503113-supplementary.pdf]

International Journal of Molecular Sciences

MDPI

Table S1. Review of methods for RNA extraction from *Gordonia* spp. and *Rhodococcus* spp.

Strain	Growth conditions	Cell disruption method	RNA extraction kit	RNA quantity	RNA integrity	Reference
G. neofelifaecis NRRL B-59395	Minimal medium with 5.7 mmol/L pyruvic acid, 1.29 mmol/L cholesterol or 1.75 mmol/L androstenedione, 30°C, 180 rpm, 36 h	No data	RNeasy mini kit (Qiagen, Hilden, Germany)	No data	No data	[41]
Gordonia sp. GONU	Mineral salt medium with 0.5 g/L mono-*n*-octyl phthalate, mono(2-ethylhexyl) phthalate, phthalic acid or succinate, 28°C, 180 rpm	In a Constant Cell Disruption System, One Shot Model, at 30 000 psi for one cycle	TRIzol	≥ 1 µg	Checked with agarose gel electrophoresis	[53]
Gordonia sp. NB4-1Y	Sulfur-free acetate medium with 0.06 mM 6:2 fluorotelomer sulfonate or 6:2 fluorotelomer sulfonamidoalkyl betaine, 150 rpm, 30°C	Fastprep-24 classic (MP Biomedical), beating at 4 m/s for 40 s a total of three times with incubation of tubes on ice for 5 min between each bead beating step	RNeasy mini kit (Qiagen, Hilden, Germany)	No data	No data	[47]
Gordonia sp. QH-11	Mineral salt medium with 500 mg/L di-*n*-butyl phthalate and phthalic acid or 1 g/L glucose, 25 or 30 h	No data	RNAprep Cell/Bacteria Kit (Tiangen, China)	≥ 1 µg	Checked with agarose gel electrophoresis	[40]
R. erythropolis CCM2595	Basal salt medium with glycerol	No particular method was used	Quick-RNA Miniprep Plus kit (Zymo Research, Irvine, CA, USA)	No data	No data	[51]
R. erythropolis D310-1	10% LB with or without 100 mg/L chlorimuron-ethyl at 28°C and 160 rpm for 96 h after acclimation on mineral salt	RiboPure Bacteria kit (Thermo Fisher Scientific, Waltham, MA, USA)		No data	Checked with agarose gel electrophoresis	[44]

	medium agar plates with 100 mg/L chlorimuron-ethyl					
R. erythropolis N186/21	Basal salt medium with 0.2% (*v*/*v*) ethanol, 28°C	Shaking cells with RNeasy and acid-washed, RNAse-free silica/ceramic material FastRNA Tube-BLUE for 20 s at a speed rating of 6 in a FastPrep FP120 shaker	RNeasy Total RNA kit (Qiagen, Hilden, Germany)	No data	Confirmed with agarose gel electrophoresis: no smears with two clear bands for 23S and 16S rRNA	[39]
R. erythropolis N186/21	LB, 28°C, overnight	Ampicillin (0.6 mg/mL) for 2–3 h, then treatment with 2 mg/mL lysozyme at room temperature for 10–15 min	RNeasy Total RNA kit (Qiagen, Hilden, Germany)	Cells remained intact and were not lysed		[39]
R. erythropolis RIA-643 infected with WC1 bacteriophage	The Middlebrook 7H9 medium for mycobacteria (contains inorganic salts, glycerol and oleic acid) with AD supplement (albumin + dextrose 10%) and $CaCl_2$ (1 mM)	Disruption in Matrix B using a Beadbeater twice for 45 s on max speed with 1 min incubation on ice in between	TRIzol solution with bromochloropropane	No data	Confirmed using a Bioanalyzer 2100 after rRNA depletion	[54]
R. imtechensis XM24D	LB at 30°C and 200 rpm, then optional addition of 0.2 mM nitrophenol to induce target genes	No data	No data	No data	No data	[50]
R. jostii RHA1	1/5 LB at 28°C, dilution, inoculation of the sterile field soil spiked with 0.2% (*w*/*w*) biphenyl, and incubation at 30°C for 24–72 h	10 g glass beads d=0.2 mm + 1 zirconia-silica ball d=15 mm + 5 g inoculated soil + 9 mL PBS + 0.5 mL 20% SDS + 0.5 mL guanidinium thiocyanate solution in a ShakeMaster Auto machine for 15 min	Phenol extraction + ethanol precipitation twice followed by purification with the Aurum Total RNA Mini Kit (Bio-Rad Laboratories, Hercules, CA, USA) and an RNase-free Sephadex G-50 quick spin column	0.2–2.0 μg	Confirmed with the 1% agarose gel electrophoresis: no smears with three clear bands for 23S rRNA, 16S rRNA and RNAs with special secondary structures are visible. Small RNAs, such	[30]

					as 5S rRNA, were lost.	
R. jostii RHA1 *R. rhodochrous* EP4	M9-Goodies with 1.0 mM 4-ethylphenol, succinate, benzoate, phenol, 3-methylphenol, 4-methylphenol, 4-propylphenol, 3,4-dimethylphenol, 2,4-dimethylphenol, 4-hydroxyphenylacetate, 4-hydroxybenzioic acid, or 0.5 mM 4-nitrophenol for 24 h	Boiling in 1M NaOH	TRIzol	≥ 1 µg	Checked with 1% agarose gel electrophoresis	[46]
R. opacus PD630	Minimal salts medium with 2 g/L glucose or 0.75 g/L phenol, and tryptic soy broth, 250 rpm, 30°C	Optional ZR BashingBead Lysis Tubes with 0.1 and 0.5 mm beads	Quick-RNA MiniPrep Plus kit (Zymo Research, Irvine, CA, USA)	≥ 1 µg	Checked with gel electrophoresis	[45]
R. opacus R7	Mineral medium M9 with 1 g/L *o*-xylene or 20 mM malate, 120 rpm, 30°C	RNA-Total RNA Mobio Isolation Kit (Qiagen Italia, Milan, Italy)		No data	RIN 8	[48]
R. opacus R7	Mineral medium M9 with 1.2% polyethylene powder or 20 mM malate	Vortexing in PowerBeadSolution (supplied with the kit) for 15 min	RNA Power Soil Total RNA Isolation Kit (Qiagen Italia, Milan, Italy) with modified purification step	No data	RIN 7.5	[49]
Rhodococcus sp. M2	LB with 10–100 ppm endosulfan, 150 rpm, 30°C, overnight	Passing cells in TRIzol several times through microtip	TRIzol	500–2000 ng/µl, negatively proportional to the endosulfan concentration	Confirmed with the 1% agarose gel electrophoresis: no smears with two clear bands for 23S rRNA and 16S rRNA	[42]
Rhodococcus sp. NJ-530	Subsequent cultivation in the Zobell 2216E medium and then in the MAMS medium containing	No particular method was used	TRIzol	No data		[52]

	1 mM dimethyl-sulfoniopropionate or a mixture of glycerol (0.45 vol. %) and glucose (0.05%), 150 rpm, 20°C					
Rhodococcus sp. TG13 (probably *R. ruber*) and TN3 (probably *R. erythropolis*)	Cells were grown in LB at 180 rpm and 30°C, or viable but nonculturable cells were obtained	No particular method was used	MICROBExpress Bacterial mRNA Enrichment kit (Thermo Fisher Scientific, Waltham, MA, USA)	≥ 20 μg	Examined with an Agilent 2100 Bioanalyzer	[43]

RIN – RNA Integrity Number defined with a Bioanalyzer capillary electrophoresis (1–5 – RNA is heavily degraded, 6–7 – some degradation of RNA, and 8–10 – RNA is intact. Removal of DNA is not specified; although, it has been performed in most studies. Cells were grown until the exponential growth phase, if otherwise is not specified. Although, no data is indicated for RNA integrity and quantity in most examples, RNA appear to be suitable for downstream steps (RT-qPCR or RNA-Seq).

International Journal of Molecular Sciences

MDPI

Table S2. Characteristics of bacterial strains

#	Strain	Source of isolation	Functional activities	**DDBJ/EMBL/GenBank acc. no**
1	*Gordonia alkanivorans* IEGM 1277 (http://www.iegmcol.ru/strains/gordonia/alkanivorans/g_alkanivorans1277.html, last accessed 29 July 2026)	Oil-polluted waste, Sosnogorsk, Komi Republic, Russia	Uses propane, *n*-hexadecane and crude oil as a sole carbon source; resistant to Pb^{2+} (20.0 mM), Ni^{2+}, Zn^{2+} (5.0 mM)	JASIRQ010000001-JASIRQ010000143 (whole genome sequence)
2	*Gordonia paraffinivorans* IEGM 735 (http://www.iegmcol.ru/strains/gordonia/paraffinivorans/g_paraffinivorans735.html, last accessed 29 July 2026)	Foam, activated sludge plant, Irvine, Scotland, UK	Uses *n*-hexadecane as a sole carbon source; resistant to Cr^{+6}, Ni^{2+}, Zn^{2+} (40.0 mM), Cd^{2+} (20.0 mM), Pb^{2+} (10.0 mM), Mo^{+6} (5.0 mM)	JAJNDG010000001-JAJNDG010000065 (whole genome sequence)
3	*Gordonia rubripertincta* IEGM 133* (http://www.iegmcol.ru/strains/gordonia/rubrip/g_rubrip133.html, last accessed 29 July 2026)	Oil-polluted soil, oilfield, Ivano-Frankovsk region, Ukraine	Uses aniline, methylbenzene, oxybenzene (phenol), phenylacetic acid, xylole; resistant to Pb^{2+} (10.0 mM)	JARWLK010000001-JARWLK010000087 (whole genome sequence)
4	*Rhodococcus cerastii* IEGM 1243 (http://www.iegmcol.ru/strains/rhodoc/cerastii/r_cerastii1243.html, last accessed 29 July 2026)	Soil, lake shore Kumnylor, Tyumen region, Russia	Uses *n*-hexadecane as a sole carbon source	JAJNDD010000001-JAJNDD010000295 (whole genome sequence)
5	*Rhodococcus pyridinivorans* IEGM 639 (http://www.iegmcol.ru/strains/rhodoc/pyridin/r_pyridin639.html, last accessed 29 July 2026)	Snow, oilfield, Perm region, Russia	Uses propane, *n*-butane, liquid *n*-alkanes (C_{11}–C_{16}) and aliphatic alcohols (ethanol, *n*-propanol, *n*-butanol) as a sole carbon source; transforms thioanisole; transforms monoterpenoid (–)-*trans*-carveol; adheres to liquid hydrocarbons (*n*-hexadecane); resistant to *n*-hexane; resistant to Cr^{+6} (40.0	JAPWIT010000001-JAPWIT010000106 (whole genome sequence)

			mM), Zn^{2+} (20.0 mM), Mo^{+6} (10.0 mM), Cd^{2+}, Pb^{2+} (5.0 mM)	
6	*Rhodococcus qingshengii*** IEGM 267 (http://www.iegmcol.ru/strains/rhodoc/qingsh/r_qingsh267.html, last accessed 29 July 2026)	Oil-polluted soil, oil-extracting enterprise, Perm region, Russia	Uses hydrocarbons and crude oil as a sole carbon source; transforms diterpenoids (dehydroabietic acid); forms cholesterol oxidase; resistant to Cr^{+6} (5.0 mM), VO^{2+} (12.5 mM), VO_4^{3-} (50.0 mM), VO_3^{-} (250.0 mM)	MRBQ01000001-MRBQ01000231 (whole genome sequence)
7	*Rhodococcus rhodochrous* IEGM 757 (http://www.iegmcol.ru/strains/rhodoc/rhodoch/r_rhod757.html, last accessed 29 July 2026)	Oil-polluted soil, Perm region, Russia	Uses *n*-hexadecane, butanol, and crude oil as a sole carbon source; resistant to Zn^{2+} (10.0 mM), Mo^{+6} (5.0 mM); transforms pentacyclic triterpenoids oleanolic and glycyrrhetinic acids (1.0 g/L); transforms monoterpenoids (–)-*cis*-carveol and (–)-L-carvone	JAJNCO010000001-JAJNCO010000163 (whole genome sequence)
8	*Rhodococcus rhodochrous* IEGM 1162 (http://www.iegmcol.ru/strains/rhodoc/rhodoch/r_rhod1162.html, last accessed 29 July 2026)	*Atriplex* rhizosphere, soil, former landfill area, Perm, Perm region, Russia	Uses *n*-butane and *n*-hexadecane as a sole carbon source; resistant to Cr^{+6}, Ni^{2+}, Zn^{2+} (40.0 mM), Mo^{+6} (10.0 mM), Cd^{2+} (5.0 mM)	MG637005.1 (16S rRNA gene)
9	*Rhodococcus rhodochrous* IEGM 1360 (http://www.iegmcol.ru/strains/rhodoc/rhodoch/r_rhod1360.html, last accessed 29 July 2026)	Moss rhizosphere, soil near ahydropost, bukhta and Tikhaya, Gukera Island, Franz Josef Land, Arkhangel'sk region, Russia	Uses *n*-hexadecane and butanol as a sole carbon source; resistant to Cr^{+6} (40.0 mM), Mo^{+6} (10.0 mM), Zn^{2+} (5.0 mM); transforms pentacyclic triterpenoids oleanolic and glycyrrhetinic acids (1.0 g/L)	JAJNCN010000001-JAJNCN010000105 (whole genome sequence)
10	*Rhodococcus rhodochrous* IEGM 1362 (http://www.iegmcol.ru/strains/rhodoc/rhodoch/r_rhod1362.html, last accessed 29 July 2026)	Peat Pal'tinskoye peat deposit, Perm region, Russia	Uses *n*-hexadecane as a sole carbon source; degrades dehydroabietic acid; resistant to Cr^{+6} (40.0 mM), Mo^{+6}, Zn^{2+} (5.0 mM); transforms monoterpenoid (–)-0.025 vol.% of (–)-isopulegol (0.025%); transforms monoterpenoids (–)-*cis*-carveol, (–)-*trans*-carveol and (–)-L-carvone	JANFQM010000001-JANFQM010000140 (whole genome sequence)

11	*Rhodococcus ruber* IEGM 231 (http://www.iegmcol.ru/strains/rhodoc/ruber/r_ruber231.html, last accessed 29 July 2026)	Water, spring, Olkhovski oil-extracting enterprise, Perm region, Russia	Uses *n*-dodecane, *n*-hexadecane, propane and *n*-butane as a sole carbon source; produces biosurfactants when growing on *n*-alkanes (C_{12}–C_{17}); degrades high-porous ceramic materials; degrades paracetamol and sodium diclofenac; forms cholesterol oxidase; resistant to Mo^{+6} (5.0 mM), VO^{2+} (25.0 mM), VO_4^{3-} (100.0 mM), VO_3^{-} (>250.0 mM), accumulates molybdenum and nickel; adheres to liquid hydrocarbons; bioremediation agent for oil-contaminated soil; resistant to 1-butanol, ethanol; produces glycolipid biosurfactant with immunomodulating properties. The dependence of the decrease in phytotoxicity of molybdenum after treatment of seeds with *Rhodococcus*-biosurfactants was revealed	CCSD01000001-CCSD01000115 (whole genome sequence)
12	*Rhodococcus ruber* IEGM 346 (http://www.iegmcol.ru/strains/rhodoc/ruber/r_ruber346.html, last accessed 29 July 2026)	Sewage, Harbin, China	Uses propane and *n*-butane as a sole carbon source; produces biosurfactants when growing on *n*-alkanes; shows pronounced emulsifying and biodegradation activity of individual hydrocarbons and oil products; bioremediation agent for oil-contaminated soil; degrades paracetamol and sodium diclofenac; resistant to Cu^{2+}, Mo^{+6}, Pb^{2+} (5.0 mM); stable under extreme acidity (pH 2–6) and salinity (2–6% NaCl). Metabolites: transforms diclofenac to fumarylacetic, fumaric and acetic acids	JAJNCM010000001-JAJNCM010000121 (whole genome sequence)
13	*Rhodococcus ruber* IEGM 560 (http://www.iegmcol.ru/strains/rhodoc/ruber/r_ruber560.html, last accessed 29 July 2026)	Sand from the depth 6.0 m, Belarus	Uses propane and *n*-butane as a sole carbon source; resistant to Cr^{+6}, Ni^{2+}, Pb^{2+}, Zn^{2+} (5.0 mM)	JASHLB010000001-JASHLB010000163 (whole genome sequence)

14	*Rhodococcus ruber* IEGM 1122* (http://www.iegmcol.ru/strains/rhodoc/ruber/r_ruber1122.html, last accessed 29 July 2026)	*Plantago major* rhizosphere, soil, former landfill area	Uses propane and *n*-butane, liquid n-alkanes (C_5–C_7, C_{11}–C_{16}) as a sole carbon source; resistant to Pb^{2+} (5.0 mM)	JASIRH010000001-JASIRH010000151 (whole genome sequence)
15	*Rhodococcus ruber* IEGM 1156 (http://www.iegmcol.ru/strains/rhodoc/ruber/r_ruber1156.html, last accessed 29 July 2026)	*Plantago* rhizosphere, former landfill area, Perm, Perm region, Russia	Uses propane, *n*-butane, and *n*-hexadecane as a sole carbon source; resistant to Mo^{+6}, Zn^{2+} (5.0 mM)	JASHLE010000001-JASHLE010000075 (whole genome sequence)
16	*Rhodococcus* sp. IEGM 1351 (http://www.iegmcol.ru/strains/rhodoc/sp/r_sp1351.html, last accessed 29 July 2026)	Pb-contaminated soil, metallurgical enterprise area, Perm, Perm region, Russia	Uses *n*-hexadecane as a sole carbon source	JASHKU010000001-JASHKU010000221 (whole genome sequence)
17	*Rhodococcus* sp. IEGM 1354 (http://www.iegmcol.ru/strains/rhodoc/sp/r_sp1354.html, last accessed 29 July 2026)	Heavy metal contaminated soil, metallurgical enterprise area, Perm, Perm region, Russia	Uses *n*-hexadecane as a sole carbon source	JASHKT010000001-JASHKT010000118 (whole genome sequence)
18	*Rhodococcus* sp. IEGM 1372 (http://www.iegmcol.ru/strains/rhodoc/sp/r_sp1372.html, last accessed 29 July 2026)	Snow, petrol station, Chusovoy, Perm region, Russia.	Uses *n*-hexadecane as a sole carbon source	JASHKS010000001-JASHKS010000104 (whole genome sequence)
19	*Rhodococcus* sp. IEGM 1379 (http://www.iegmcol.ru/strains/rhodoc/sp/r_sp1379.html, last accessed 29 July 2026)	Oil-polluted soil, petroleum station, Perm, Perm region, Russia.	Uses *n*-hexadecane as a sole carbon source	JASHKR010000001-JASHKR010000230 (whole genome sequence)
20	*Rhodococcus* sp. IEGM 1381 (http://www.iegmcol.ru/strains/rhodoc/sp/r_sp1381.html, last accessed 29 July 2026)	Oil-polluted soil, petroleum station, Perm, Perm region, Russia	Uses *n*-hexadecane as a sole carbon source	JASHKQ010000001-JASHKQ010000043 (whole genome sequence)
21	*Rhodococcus* sp. IEGM 1404* (http://www.iegmcol.ru/strains/rhodoc/sp/r_sp1404.html, last accessed 29 July 2026)	Bottom sediments, Severnaya Zemlya, Krasnoyarsk region, Russia	Uses *n*-hexadecane as a sole carbon source	JASHKP010000001-JASHKP010000055 (whole genome sequence)

*Species names are different on the site of the IEGM Collection (http://www.iegmcol.ru/) and in GenBank: Additional confirmation with phenotypic tests according to the polyphasic taxonomy approach is required for this strain.
***Rhodococcus qingshengii* is a synonym of *Rhodococcus erythropolis* (https://lpsn.dsmz.de/species/rhodococcus-qingshengii, last accessed 29 July 2026).

https://doi.org/10.3390/xxxxx

International Journal of Molecular Sciences

MDPI

Table S3. Substrates and growth conditions

Substrate / Additive	Supplier (purity)	Concentration	Medium	Notes
**Carbohydrates**				
D-glucose	Sigma-Aldrich (≥ 98%)	1.0 or 5.0 g/L	RS, agar K	Growth substrate
**Alcohols**				
Glycerol	Sigma-Aldrich, Burlington, MA, USA (≥ 98%)	0.1 vol.%	RS	Growth substrate
**Organic acids**				
Ammonium acetate	Sigma-Aldrich, Burlington, MA, USA (≥ 98%)	20.0 g/L	K	Growth substrate
**Gaseous alkanes**				
Propane	NPP Sintez, LLC, Perm, Russia (≥ 99.5%)	Atmosphere propane : air 1 : 4	Agar K	Growth substrate, cultivation in a closed desiccator
n-Butane	Sigma-Aldrich, Burlington, MA, USA (≥ 98%)	Atmosphere *n*-butane : air 1 : 4	Agar K	Growth substrate, cultivation in a closed desiccator
**Liquid alkanes**				
n-Pentane	Sigma-Aldrich, Burlington, MA, USA (≥ 98%)	0.1 vol.%	RS	Growth substrate
n-Hexane	Sigma-Aldrich, Burlington, MA, USA (≥ 98%)	0.1 vol.%	RS	Growth substrate
n-Heptane	Sigma-Aldrich, Burlington, MA, USA (≥ 98%)	0.1 vol.%	RS	Growth substrate
n-Octane	Sigma-Aldrich, Burlington, MA, USA (≥ 98%)	0.1 vol.%	RS	Growth substrate
n-Nonane	Sigma-Aldrich, Burlington, MA, USA (≥ 98%)	0.1 vol.%	RS	Growth substrate
n-Decane	Sigma-Aldrich, Burlington, MA, USA (≥ 98%)	0.1 vol.%	RS	Growth substrate
n-Undecane	Sigma-Aldrich, Burlington, MA, USA (≥ 98%)	0.1 vol.%	RS	Growth substrate
n-Dodecane	Sigma-Aldrich, Burlington, MA, USA (≥ 98%)	0.1 vol.%	RS	Growth substrate
n-Tridecane	Sigma-Aldrich, Burlington, MA, USA (≥ 98%)	0.1 vol.%	RS	Growth substrate
n-Tetradecane	Sigma-Aldrich, Burlington, MA, USA (≥ 98%)	0.1 vol.%	RS	Growth substrate
n-Pentadecane	Sigma-Aldrich, Burlington, MA, USA (≥ 98%)	0.1 vol.%	RS	Growth substrate
n-Hexadecane	Sigma-Aldrich, Burlington, MA, USA (≥ 98%)	0.1 vol.%	RS	Growth substrate

Pristane	Sigma-Aldrich, Burlington, MA, USA (≥ 98%)	0.1 vol.%	RS	Growth substrate
**Monoaromatic hydrocarbons**				
Benzene	Sigma-Aldrich, Burlington, MA, USA (≥ 98%)	0.1 vol.%	RS, agar K	Growth substrate
o-Xylene	Sigma-Aldrich, Burlington, MA, USA (≥ 98%)	0.1 vol.%	RS, agar K	Growth substrate
**Polycyclic aromatic hydrocarbons**				
Naphthalene	Sigma-Aldrich, Burlington, MA, USA (≥ 98%)	1.0 g/L	Agar K	Growth substrate
Phenanthrene	Sigma-Aldrich, Burlington, MA, USA (≥ 98%)	1.0 g/L	Agar K	Growth substrate
**Oil**				
Used frying oil	Local KFC restaurant, Perm, Russia	0.05 or 0.1 vol.%	RS	Growth substrate
**Terpenoids**				
(−)-Isopulegol	Synthesized by the Chemistry faculty of the Novosibirsk State University, Novosibirsk, Russia	0.025 vol.%	RS, LB	Growth substrate, which is better oxidized in the presence of yeast extract. Dissolved in DMSO (1 mg in 10 μL). In RS, it is used along with yeast extract (0.1 g/L), the transformation was conducted by resting cells, and halloysite nanotubes (0.1 g/L) were optionally used.
Oleanolic acid	Thermo Fisher Scientific, Waltham, MA, USA (≥98%)	0.5 or 1.0 g/L	RS	Growth substrate, which is better oxidized in the presence of yeast extract. Dissolved in DMSO (1 mg in 10 μL), used along with yeast extract (0.1 g/L), transformation by resting cells
Glycyrrhetinic acid	TCI, Antwerpen, Belgium (≥98%)	0.5 or 1.0 g/L	RS	Growth substrate, which is better oxidized in the presence of yeast extract. Dissolved in DMSO (1 mg in 10 μL), used along with yeast extract (0.1 g/L),

				transformation by resting cells
		**Non steroid anti-inflammatory drugs (NSAIDs)**		
Ketoprofen	BLD Pharmatech Ltd, Shanghai, China (≥ 99.99%)	0.1 vol.%	RS	Dissolved in ethanol (0.01 g in 500 μL), a co-metabolic substrate used with 0.1 vol.% of *n*-hexadecane as a growth substrate
Meloxicam	BLD Pharmatech Ltd, Shanghai, China (≥ 99.99%)	0.01 g/L	RS	Dissolved in ethanol (0.01 g in 500 μL), a co-metabolic substrate used with 0.1 vol.% of *n*-hexadecane as a growth substrate
Naproxen	BLD Pharmatech Ltd, Shanghai, China (≥ 99.99%)	0.01 g/L	RS	Dissolved in ethanol (0.01 g in 500 μL), a co-metabolic substrate used with 5 g/L D-glucose as a growth substrate
Diclofenac + Ibuprofen + Naproxen	BLD Pharmatech Ltd, Shanghai, China (≥ 99.99%)	0.01 g/L each	RS	Dissolved in ethanol (0.01 g in 500 μL), co-metabolic substrates used with 5 g/L D-glucose as a growth substrate
		**Heavy metals**		
$HgCl_2$	Reachim, Russia (≥ 99.99%)	10.9 or 86.9 mg/L	LB	A toxic inorganic additive

LB is not included in this Table separately as it is not a medium supplement but rather a base like K and RS.

Table S4. Electropherograms of isolated RNAs

#	Strain	Electropherogram	Cell lysis method	RNA extraction method	Growth conditions
1	*R. ruber* IEGM 231	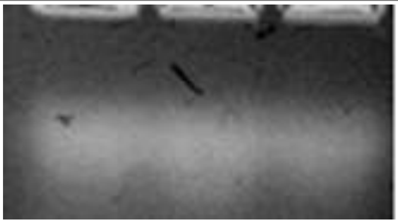	Without lysis	TRIzol™ LS Reagent, Invitrogen, Carlsbad, CA, USA	LB for 24 h
2	*R. ruber* IEGM 231	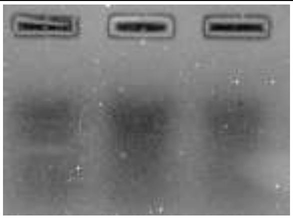	Without lysis	TRIzol™ LS Reagent, Invitrogen, Carlsbad, CA, USA	LB for 24 h
3	*R. ruber* IEGM 231	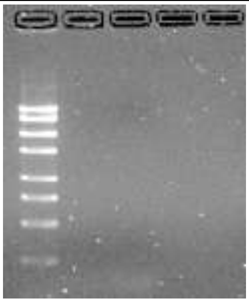	Freezing-thawing, three cycles −20°C/96°C	TRIzol™ LS Reagent, Invitrogen, Carlsbad, CA, USA	From left to right: 1: DNA ladder (250 bp–3 kb); 2–5: LB for 24 h
4	*R. ruber* IEGM 231	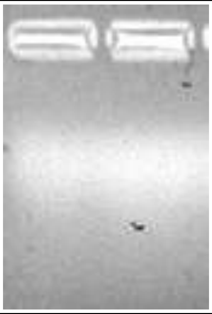	Lysozyme (40 mg/mL), 37°C for 1 h	TRIzol™ LS Reagent, Invitrogen, Carlsbad, CA, USA	LB for 48 h
5	*R. ruber* IEGM 231	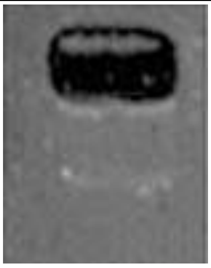	Lysozyme (40 mg/mL), 37°C for 1 h + 10% SDS	TRIzol™ LS Reagent, Invitrogen, Carlsbad, CA, USA	LB for 60 h

6	*R. ruber* IEGM 231	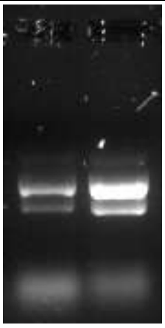	Lysozyme (40 mg/mL), 37°C for 1 h + 10% SDS	RNA Solo, Evrogen, Moscow, Russia	From left to right: 1: RS + 0.1 vol.% ketoprofen for 48 h; 2: LB for 48 h
7	*R. ruber* IEGM 231	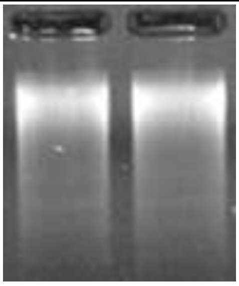	Metal beads (Ø=1 mm; quantity=10 beads; v=5 m/s; t=2 min)	TRIzol™ LS Reagent, Invitrogen, Carlsbad, CA, USA	RS + 0.1 vol.% *n*-hexadecane for 48 h
8	*R. ruber* IEGM 231	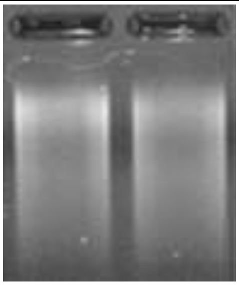	Metal beads (Ø=3 mm; quantity=10 beads; v=5 m/s; t=2 min)	TRIzol™ LS Reagent, Invitrogen, Carlsbad, CA, USA	RS + 0.1 vol.% *n*-hexadecane for 48 h
9	*R. ruber* IEGM 231	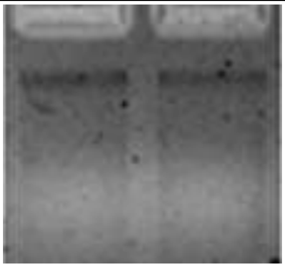	Metal beads (Ø=1 mm; quantity=10 beads; v=5 m/s; t=2 min)	RNeasy Mini Kit, Qiagen, Hilden, Germany	From left to right: 1: agar K, propane : air 1:4 for 48 h; 2: agar K, *n*-butane: air 1:4 for 48 h
10	*R. ruber* IEGM 231	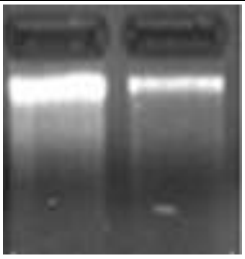	Metal beads (Ø=1 mm; quantity=10 beads; v=5 m/s; t=2 min)	RNeasy Mini Kit, Qiagen, Hilden, Germany	From left to right: 1: RS + 0.1 vol.% *n*-tetradecane for 48 h; 2: RS + 0.1 vol.% *n*-pentadecane for 48 h
11	*R. ruber* IEGM 231	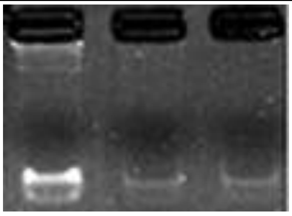	Metal beads (Ø=1 mm; quantity=10 beads; v=5 m/s; t=2 min)	RNeasy Mini Kit, Qiagen, Hilden, Germany	From left to right: 1: RS + 0.1 vol.% *n*-hexadecane for 48 h; 2: RS + 0.1 vol.% *n*-undecane for 48 h; 3: RS + 0.1 vol.% pristane for 48 h

12	*R. ruber* IEGM 231	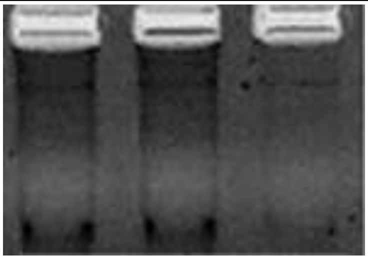	Metal beads (Ø=1 mm; quantity=10 beads; v=5 m/s; t=2 min)	RNeasy Mini Kit, Qiagen, Hilden, Germany	From left to right: 1: RS + 0.1 vol.% *n*-nonane for 48 h; 2: RS + 0.1 vol.% *n*-decane for 48 h; 3: RS + 0.1 vol.% *n*-dodecane for 48 h
13	*R. ruber* IEGM 231	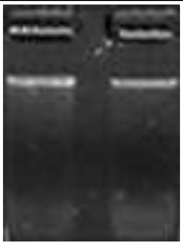	Metal beads (Ø=1 mm; quantity=10 beads; v=5 m/s; t=2 min)	RNeasy Mini Kit, Qiagen, Hilden, Germany	From left to right: 1: RS + 0.1 vol.% *n*-tridecane for 48 h; 2: RS + 0.1 vol.% *n*-pentane for 48 h
14	*R. ruber* IEGM 231	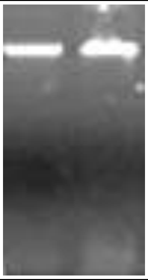	Metal beads (Ø=1 mm; quantity=10 beads; v=5 m/s; t=2 min)	RNeasy Mini Kit, Qiagen, Hilden, Germany	From left to right: 1: RS + 0.1 vol.% *n*-hexane for 48 h; 2: RS + 0.1 vol.% *n*-heptane for 48 h
15	*R. ruber* IEGM 231	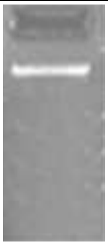	Metal beads (Ø=1 mm; quantity=10 beads; v=5 m/s; t=2 min)	RNeasy Mini Kit, Qiagen, Hilden, Germany	RS + 0.1 vol.% *n*-octane for 48 h
16	*R. ruber* IEGM 231	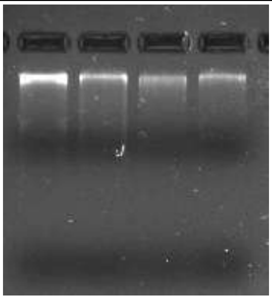	Metal beads (Ø=1 mm; quantity=10 beads; v=5 m/s; t=2 min)	RNeasy Mini Kit, Qiagen, Hilden, Germany	From left to right: 1: LB for 48 h; 2: RS+ 1.0 g/L glucose for 48 h; 3: agar K + 1.0 g/L naphthalene for 48 h; 4: agar K + 1.0 g/L phenanthrene for 48 h
17	*R. ruber* IEGM 231	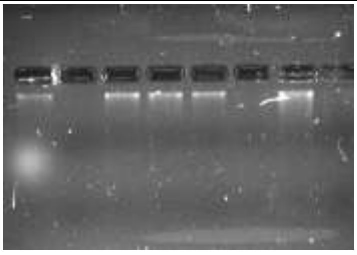	Metal beads (Ø=1 mm; quantity=10 beads; v=5 m/s; t=2 min)	RNeasy Mini Kit, Qiagen, Hilden, Germany	From left to right: 1-2: RS + 0.1 vol.% *n*-nonane for 48 h; 3-4: RS + 0.1 vol.% *o*-xylene for 48 h; 5-6: RS + 0.1 vol.% glycerol for 48 h; 7-8: LB for 48 h

18	*R. ruber* IEGM 231	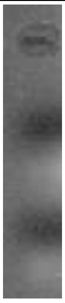	Metal beads (Ø=1 mm; quantity=10 beads; v=5 m/s; t=2 min)	RNA Solo, Evrogen, Moscow, Russia	LB for 48 h
19	*R. ruber* IEGM 231	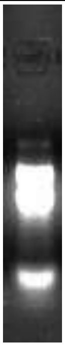	Lysozyme (40 mg/mL), 37°C for 1 h + 10% SDS	RNA Solo, Evrogen, Moscow, Russia	LB for 48 h
20	*R. ruber* IEGM 231	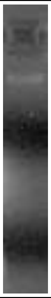	Metal beads (Ø=1 mm; quantity=10 beads; v=5 m/s; t=2 min)	RNA Solo without DNase purification, Evrogen, Moscow, Russia	LB for 48 h
21	*R. ruber* IEGM 231	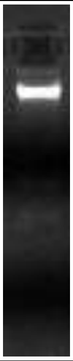	Lysozyme (40 mg/mL), 37°C for 1 h + 10% SDS	RNA Solo without DNase purification, Evrogen, Moscow, Russia	LB for 48 h
22	*R. ruber* IEGM 231	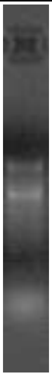	Metal beads (Ø=1 mm; quantity=10 beads; v=5 m/s; t=2 min)	RNA Solo, Evrogen, Moscow, Russia	RS + 0.1 vol.% ketoprofen for 48 h

23	*R. ruber* IEGM 231	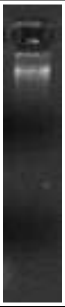	Metal beads (Ø=1 mm; quantity=10 beads; v=5 m/s; t=2 min)	RNA Solo without DNase purification, Evrogen, Moscow, Russia	RS + 0.1 vol.% ketoprofen for 48 h
24	*R. ruber* IEGM 231	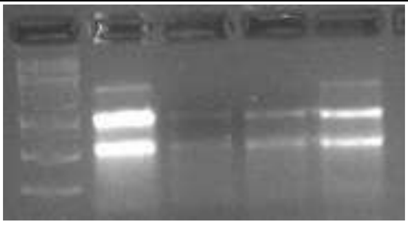	Lysozyme (40 mg/mL), 37°C for 1 h + 10% SDS	RNA Solo, Evrogen, Moscow, Russia	From left to right: 1: DNA ladder (250 bp–10 kb); 2-3: LB for 48 h; 4-5: RS + 0.1 vol.% *n*-hexadecane for 48 h
25	*R. ruber* IEGM 231	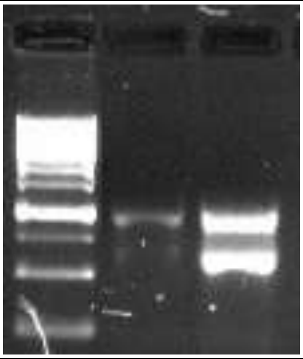	Lysozyme (40 mg/mL), 37°C for 1 h + 10% SDS	RNA Solo, Evrogen, Moscow, Russia	From left to right: 1: DNA ladder (250 bp–10 kb); 2: RS + 0.1 vol.% *n*-hexadecane; 3: LB for 48 h
26	*R. ruber* IEGM 231	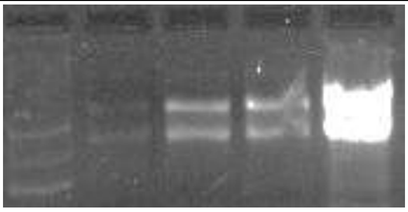	Lysozyme (40 mg/mL), 37°C for 1 h + 10% SDS	RNA Solo, Evrogen, Moscow, Russia	From left to right: 1: DNA ladder (250 bp–10 kb); 2: RS + 0.1 vol.% *n*-decane for 48 h; 3: RS + 0.1 vol.% *n*-dodecane for 48 h; 4: RS + 0.1 vol.% *n*-hexadecane for 48 h; 5: LB for 48 h
27	*R. ruber* IEGM 231	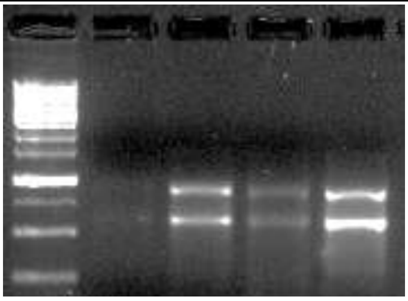	Lysozyme (40 mg/mL), 37°C for 1 h + 10% SDS	RNA Solo, Evrogen, Moscow, Russia	From left to right: 1: DNA ladder (250 bp–10 kb); 2: RS + 0.1 vol.% *n*-undecane for 48 h; 3: RS + 0.1 vol.% *n*-dodecane for 48 h; 4: RS + 0.1 vol.% *n*-hexadecane for 48 h; 5: LB for 48 h

28	*R. ruber* IEGM 231	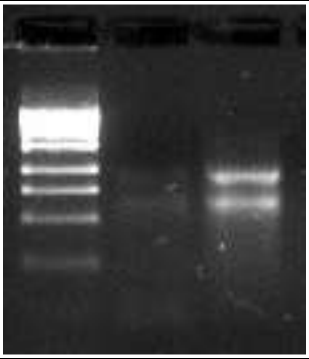	Lysozyme (40 mg/mL), 37°C for 1 h + 10% SDS	RNA Solo, Evrogen, Moscow, Russia	From left to right: 1: DNA ladder (250 bp–10 kb); 2: RS + 0.1 vol.% *n*-dodecane for 48 h; 3: RS medium + 0.1 vol.% *n*-hexadecane for 48 h
29	*R. ruber* IEGM 231	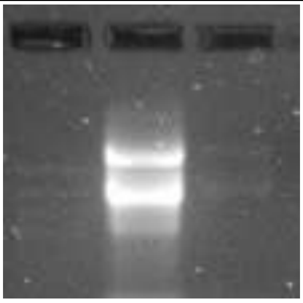	Lysozyme (40 mg/mL), 37°C for 1 h + 10% SDS	RNA Solo, Evrogen, Moscow, Russia	From left to right: 1: DNA ladder (250 bp–3 kb); 2: LB for 48 h; 3: RS + 1 g/L glucose for 48 h
30	*R. ruber* IEGM 231	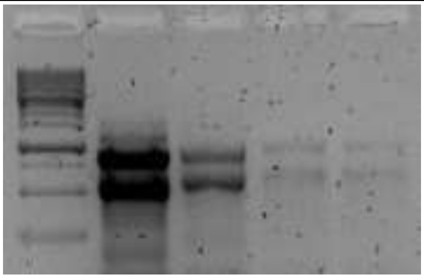	Lysozyme (40 mg/mL), 37°C for 1 h + 10% SDS	RNA Solo, Evrogen, Moscow, Russia	From left to right: 1: DNA ladder (250 bp–10 kb); 2: LB for 48 h; 3: RS + 1.0 g/L glucose for 48 h; 4: RS + 0.1 vol.% *n*-hexadecane for 48 h; 5: RS + 0.1 vol.% *n*-dodecane for 48 h
31	*R. ruber* IEGM 231	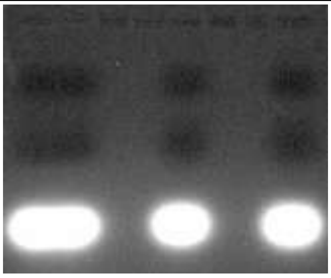	Metal beads (Ø=1 mm; quantity=40 beads; v=5 m/s; t=10 min)	TRIzol™ LS Reagent, Invitrogen, Carlsbad, CA, USA	RS + 0.1 vol.% *n*-hexadecane for 48 h
32	*R. ruber* IEGM 231	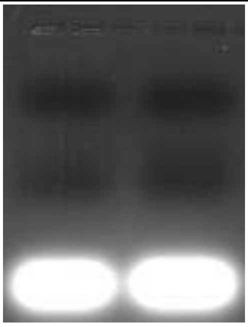	Metal beads (Ø=1 mm; quantity=40 beads; v=5 m/s; t=10 min)	TRIzol™ LS Reagent, Invitrogen, Carlsbad, CA, USA	RS + 0.1 vol.% *n*-hexadecane for 48 h

33	*R. ruber* IEGM 231	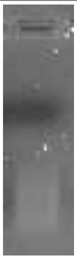	Metal beads (Ø=1 mm; quantity=40 beads; v=5 m/s; t=10 min)	TRIzol™ LS Reagent, Invitrogen, Carlsbad, CA, USA	RS + 0.1 vol.% *n*-hexadecane for 48 h
34	*R. ruber* IEGM 231	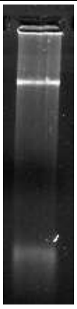	Metal beads (Ø=1 mm; quantity=40 beads; v=5 m/s; t=10 min)	TRIzol™ LS Reagent, Invitrogen, Carlsbad, CA, USA	LB for 48 h
35	*R. ruber* IEGM 231	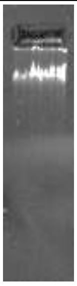	Lysozyme (40 mg/mL), 37°C for 1 h + 10% SDS	TRIzol™ LS Reagent, Invitrogen, Carlsbad, CA, USA	RS + 0.1 vol.% *n*-hexadecane for 48 h
36	*R. ruber* IEGM 231	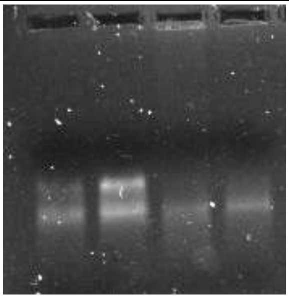	Metal beads (Ø=1 mm; quantity=40 beads; v=5 m/s; t=10 min); and the homogenate pH was adjusted (from left to right) with 1 M $NaHCO_3$ (1–2) and 1 M NaOH (3–4)	TRIzol™ LS Reagent, Invitrogen, Carlsbad, CA, USA	RS + 0.1 vol.% *n*-hexadecane for 48 h
37	*R. ruber* IEGM 231	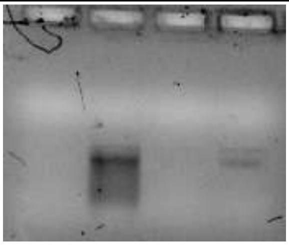	Metal beads (Ø=1 mm; quantity=40 beads; v=5 m/s; t=10 min); and the homogenate pH was adjusted (from left to right) with 1 M $NaHCO_3$ (1–2) and 1M NaOH (3–4)	TRIzol™ LS Reagent, Invitrogen, Carlsbad, CA, USA	RS + 0.1 vol.% *n*-hexadecane for 48 h

38	*R. ruber* IEGM 231	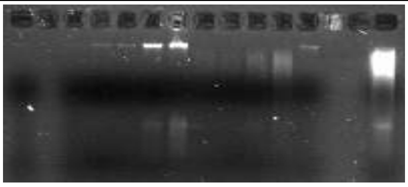	Metal beads (Ø=1 mm; quantity=40 beads; v=5 m/s; t=10 min)	RNeasy Mini Kit, Qiagen, Hilden, Germany	LB for 48 h (four biological replicates with 3–4 technical replicates are shown)
39	*R. ruber* IEGM 231	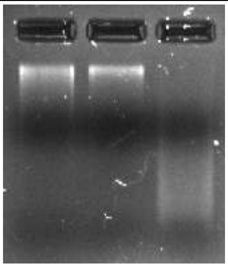	From left to right: 1: metal beads (Ø=3 mm; quantity=10 beads; v=6 m/s; t=1 min); 2-3: metal beads (Ø=1 mm; quantity=10 beads; v=6 m/s; t=1 min). Cells were resuspended in 4M guanidine isothiocyanate (1-2)	RNeasy Mini Kit, Qiagen, Hilden, Germany	RS + 0.1 vol.% *n*-hexadecane for 48 h
40	*R. ruber* IEGM 231	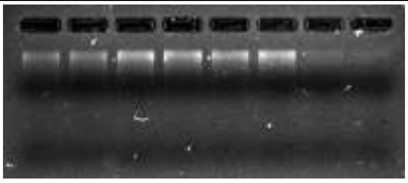	Metal beads (Ø=1 mm; quantity=10 beads; v=5 m/s; t=2 min). Cells were resuspended in 4 M guanidinium thiocyanate	RNeasy Mini Kit, Qiagen, Hilden, Germany	From left to right: 1-2: RS + 0.1 vol.% pristane for 48 h; 3-4: RS + 0.1 vol.% *n*-undecane for 48 h; 5-6: RS + 0.1 vol.% benzene for 48 h; 7-8: RS + 0.1 vol.% *o*-xylene for 48 h
41	*R. ruber* IEGM 231	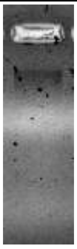	Metal beads (Ø=1 mm; quantity=10 beads; v=5 m/s; t=2 min). Cells were resuspended in 4 M guanidinium thiocyanate	RNeasy Mini Kit, Qiagen, Hilden, Germany	RS + 1.0 g/L glucose for 48 h

42	*R. ruber* IEGM 231	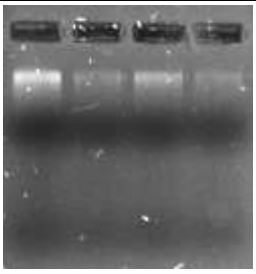	Metal beads (Ø=1 mm; quantity=10 beads; v=5 m/s; t=2 min). Cells were re-suspended in 4 M guanidinium thiocyanate.	RNeasy Mini Kit, Qiagen, Hilden, Germany	From left to right: 1-2: RS + 0.1 vol.% *n*-undecane for 48 h; 3-4: RS + 5.0 g/L glucose for 48 h
43	*R. ruber* IEGM 231	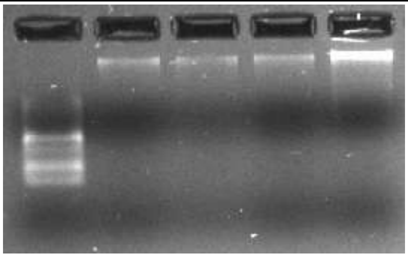	Metal beads (Ø=1 mm; quantity=10 beads; v=5 m/s; t=2 min). Cells were re-suspended in 4 M guanidinium thiocyanate (2-4 from left to right)	RNeasy Mini Kit, Qiagen, Hilden, Germany	From left to right: 1: RNA ladder (50–1000 nt); 2-3: RS + 0.1 vol.% *n*-undecane for 48 h; 4: RS + 5.0 g/L glucose 48 h; 5: LB for 48 h
44	*R. ruber* IEGM 231	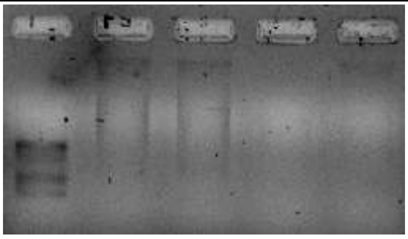	Metal beads (Ø=1 mm; quantity=10 beads; v=5 m/s; t=2 min). Cells were re-suspended in 4 M guanidinium thiocyanate	RNeasy Mini Kit, Qiagen, Hilden, Germany	From left to right: 1: RNA ladder (50–1000 nt); 2-3: RS medium + 0.1 vol.% of *n*-hexadecane for 48 h; 4-5: RS + 0.1 vol.% glycerol for 48 h
45	*R. ruber* IEGM 231	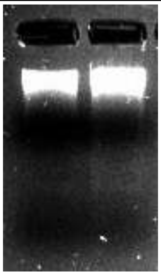	Metal beads (Ø=1 mm; quantity=15 beads; v=5 m/s; t=2 min)	RNeasy Mini Kit, Qiagen, Hilden, Germany	RS + 0.1 vol.% *n*-hexadecane for 48 h
46	*R. ruber* IEGM 231	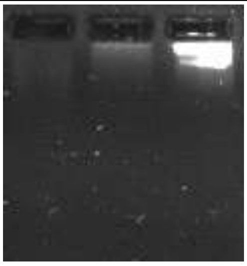	Metal beads (Ø=1 mm; quantity=10 beads; v=5 m/s; t=2 min)	RNeasy Mini Kit, Qiagen, Hilden, Germany	From left to right: 1: RS + 0.1 vol.% *n*-nonane for 48 h; 2: RS + 0.1 vol.% *n*-decane for 48 h; 3: RS + 0.1 vol.% *n*-dodecane for 48 h

47	*R. ruber* IEGM 231	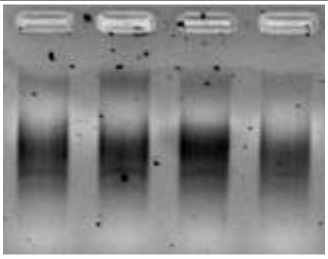	Metal beads (Ø=1 mm; quantity=10 beads; v=5 m/s; t=2 min)	RNeasy Mini Kit, Qiagen, Hilden, Germany	Agar K, propane : air 1 : 4 for 48 h
48	*R. ruber* IEGM 231	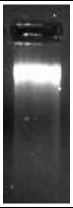	Metal beads (Ø=1 mm; quantity=10 beads; v=5 m/s; t=2 min)	RNeasy Mini Kit, Qiagen, Hilden, Germany	Agar K, propane : air 1 : 4 for 48 h
49	*R. ruber* IEGM 231	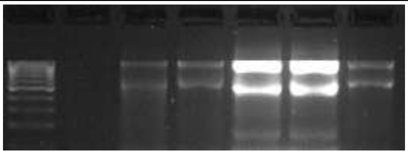	Lysozyme (40 mg/mL), 37°C for 1 h + 10% SDS	RNA Solo, Evrogen, Moscow, Russia	From left to right: 1: DNA ladder (250 bp–3 kb); 2-4: RS + 0.1 vol.% *n*-hexadecane for 120 h; 5-6: LB for 120 h; 7: RS + 1.0 g/L glucose for 120 h
50	*R. ruber* IEGM 231	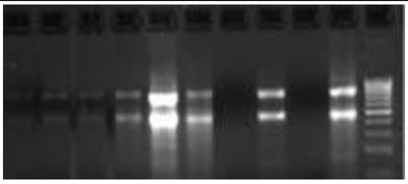	Lysozyme (40 mg/mL), 37°C for 1 h + 10% SDS	RNA Solo, Evrogen, Moscow, Russia	From left to right: 1-3: RS + 0.1 vol.% of *n*-dodecane for 48 h; 4-6: RS + 0.1 vol.% *n*-hexadecane for 48 h; 7-9: RS + 0.05 vol.% used frying oil for 48 h; 10: RS + 1.0 g/L glucose for 48 h; 11: DNA ladder (250 bp–3 kb)

51	*R. rhodochrous* IEGM 1362	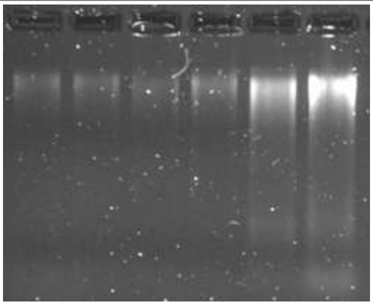	Metal beads (Ø=1 mm; quantity=10 beads; v=5 m/s; t=2 min)	RNeasy Mini Kit, Qiagen, Hilden, Germany	From left to right: 1: RS + 0.025 vol.% (–)-isopulegol+ 0.1 g/L of yeast extract for 48 h (resting cells); 2: RS + 0.025 vol.% (–)-isopulegol + 0.1 g/L yeast extract + 0.1 vol.% halloysite for 48 h (resting cells); 3: RS + 0.1 g/L yeast extract + 0.1 vol.% halloysite for 48 h (resting cells); 4: RS + 0.1 g/L yeast extract for 48 h (resting cells); 5: LB for 48 h; 6: 2xLB for 48 h
52	*R. rhodochrous* IEGM 1362	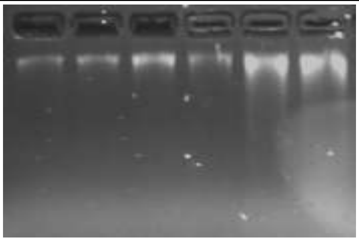	Metal beads (Ø=1 mm; quantity=10 beads; v=5 m/s; t=2 min)	RNeasy Mini Kit, Qiagen, Hilden, Germany	From left to right: 1-2: RS + 0.025 vol.% (–)-isopulegol + 0.1 g/L yeast extract for 48 h (resting cells); 3-4: RS + 0.1 g/L of yeast extract for 48 h (resting cells); 5-6: LB for 48 h
53	*R. rhodochrous* IEGM 1362	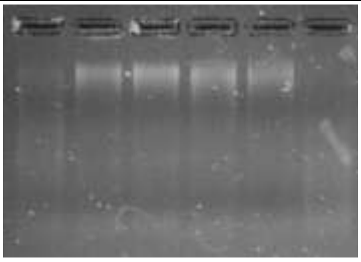	Metal beads (Ø=1 mm; quantity=10 beads; v=5 m/s; t=2 min)	RNeasy Mini Kit, Qiagen, Hilden, Germany: 1,6: with DNase purification; 2–5: no DNase purification	From left to right: 1-3: RS + 0.025 vol.% (–)-isopulegol + 0.1 g/L yeast extract for 48 h (resting cells); 4-6: RS + 0.1 g/L yeast extract for 48 h (resting cells)

54	*R. rhodochrous* IEGM 1362	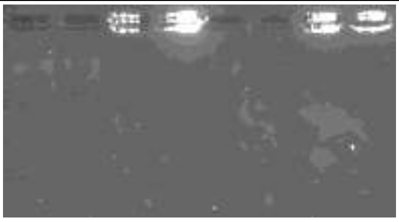	Metal beads (Ø=1 mm; quantity=10 beads; v=5 m/s; t=2 min)	From left to right: 1-2, 5-6: RNeasy Mini Kit, Qiagen, Hilden, Germany, with DNase purification; 3-4, 7-8: RNeasy Mini Kit, Qiagen, Hilden, Germany without DNase purification	From left to right: 1: RS + 0.1 g/L yeast extract for 48 h (resting cells); 2: RS + 0.025 vol.% (–)-isopulegol + 0.1 g/L of yeast extract for 48 h (resting cells); 3-4: RS + 0.025 vol.% (–)-isopulegol + 0.1 g/L yeast extract for 48 h (resting cells); 5: RS + 0.1 g/L yeast extract for 48 h (resting cells); 6: RS + 0.025 vol.% (–)-isopulegol + 0.1 g/L yeast extract for 48 h (resting cells); 7: RS + 0.1 g/L yeast extract for 48 h (resting cells); 8: RS + 0.025 vol.% (–)-isopulegol + 0.1 g/L yeast extract for 48 h (resting cells)
55	*R. rhodochrous* IEGM 1362	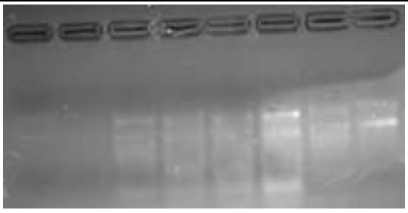	Lysozyme (40 mg/mL), 37°C for 1 h	RNA Solo, Evrogen, Moscow, Russia	From left to right: 1-2: RS + 0.025 vol.% (–)-isopulegol + 0.1 g/L yeast extract + 0.1 vol.% halloysite for 120 h (resting cells); 3-4: RS + 0.1 g/L yeast extract + 0.1 vol.% halloysite for 120 h (resting cells); 5-6: LB + 0.025 vol.% (–)-isopulegol for 120 h; 7-8: LB for 120 h

56	*R. rhodochrous* IEGM 1362	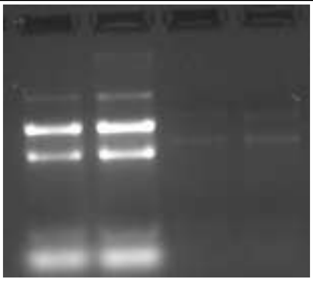	Lysozyme (40 mg/mL), 37°C for 1 h	RNA Solo, Evrogen, Moscow, Russia	From left to right: 1-2: LB + 0.025 vol.% (–)-isopulegol for 120 h; 3-4: LB for 120 h
57	*R. rhodochrous* IEGM 1362	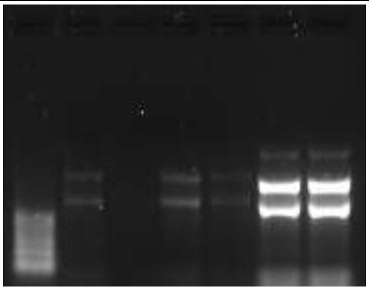	Lysozyme (40 mg/mL), 37°C for 1 h	RNA Solo, Evrogen, Moscow, Russia	From left to right: 1: RNA ladder (50–1000 nt); 2-4: RS + 0.1 g/L yeast extract + 0.1 vol.% halloysite for 120 h (resting cells); 5-7: RS + 0.025 vol.% (–)-isopulegol + 0.1 g/L yeast extract + 0.1 vol.% halloysite for 120 h (resting cells)
58	*R. rhodochrous* IEGM 1362	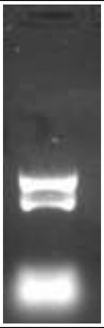	Lysozyme (40 mg/mL), 37°C for 1 h	RNA Solo, Evrogen, Moscow, Russia	LB for 120 h
59	*R. qingshengii* IEGM 267	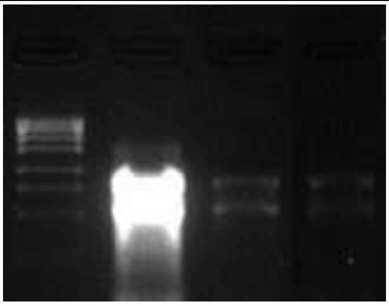	Lysozyme (40 mg/mL), 37°C for 1 h + 10% SDS	RNA Solo, Evrogen, Moscow, Russia	From left to right: 1: DNA ladder (250 bp–3 kb); 2: LB for 48 h; 3: RS + 1.0 g/L glucose for 48 h; 4: RS + 0.1 vol.% *n*-hexadecane for 48 h

60	*R. qingshengii* IEGM 267	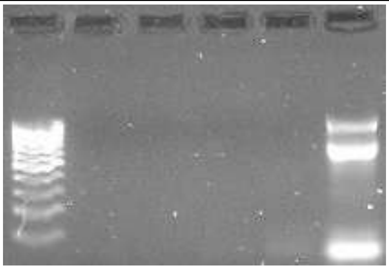	Lysozyme (40 mg/mL), 37°C for 1 h + 10% SDS	RNA Solo, Evrogen, Moscow, Russia	From left to right: 1: DNA ladder (250 bp–3 kb); 2-4: RS + 0.1 vol.% used frying oil for 48 h; 5: RS + 1.0 g/L glucose for 48 h; 6: RS + 0.1 vol.% *n*-hexadecane for 48 h
61	*R. qingshengii* IEGM 267	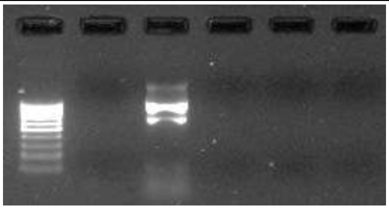	Lysozyme (40 mg/mL), 37°C for 1 h + 10% SDS	RNA Solo, Evrogen, Moscow, Russia	From left to right: 1: DNA ladder (250 bp–3 kb); 2: RS + 1.0 g/L glucose for 48 h; 3: LB for 48 h; 4-6: RS + 0.05 vol.% used frying oil for 48 h
62	*R. qingshengii* IEGM 267	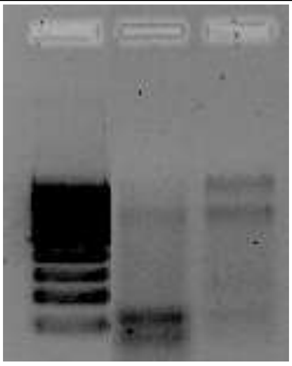	Lysozyme (40 mg/mL), 37°C for 1 h + 10% SDS	RNA Solo, Evrogen, Moscow, Russia	From left to right: 1: DNA ladder (250 bp–3 kb); 2-3: RS + 0.05 vol.% used frying oil for 48 h, cells were pre-washed with 0.1% Tween 80 and 0.5% NaCl
63	*Rhodococcus* sp. IEGM 1354	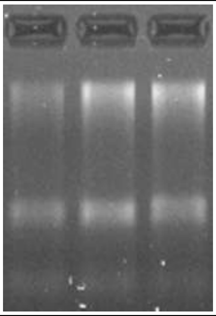	Metal beads (Ø=1 mm; quantity=10 beads; v=5 m/s; t=2 min)	RNeasy Mini Kit, Qiagen, Hilden, Germany	Agar K, propane : air 1 : 4 for 14 days
64	*Rhodococcus* sp. IEGM 1354	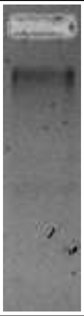	Metal beads (Ø=1 mm; quantity=10 beads; v=5 m/s; t=2 min)	RNeasy Mini Kit, Qiagen, Hilden, Germany	K without growth substrate for 14 days (oligotrophic conditions)

65	*Rhodococcus* sp. IEGM 1354	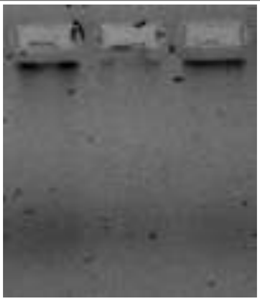	Metal beads (Ø=1 mm; quantity=10 beads; v=5 m/s; t=2 min)	RNeasy Mini Kit, Qiagen, Hilden, Germany	From left to right: 1: LB for 7 days 2: K + 20.0 g/L ammonium acetate for 14 days; 3: K + 1.0 g/L glucose for 14 days
66	*Rhodococcus* sp. IEGM 1354	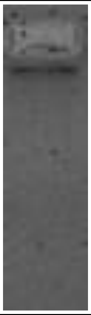	Metal beads (Ø=1 mm; quantity=10 beads; v=5 m/s; t=2 min)	RNeasy Mini Kit, Qiagen, Hilden, Germany	LB agar for 14 days
67	*Rhodococcus* sp. IEGM 1379	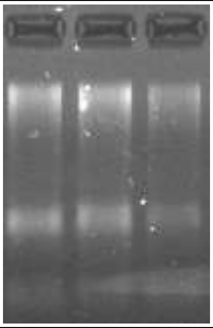	Metal beads (Ø=1 mm; quantity=10 beads; v=5 m/s; t=2 min)	RNeasy Mini Kit, Qiagen, Hilden, Germany	Agar K, propane : air 1 : 4 for 14 days
68	*Rhodococcus* sp. IEGM 1379	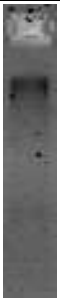	Metal beads (Ø=1 mm; quantity=10 beads mL; v=5 m/s; t=2 min)	RNeasy Mini Kit, Qiagen, Hilden, Germany	K without growth substrate for 14 days (oligotrophic conditions)
69	*Rhodococcus* sp. IEGM 1379	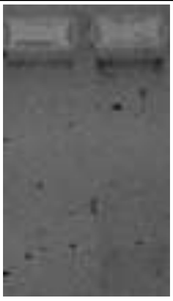	Metal beads (Ø=1 mm; quantity=10 beads; v=5 m/s; t=2 min)	RNeasy Mini Kit, Qiagen, Hilden, Germany	From left to right: 1: K + 20.0 g/L ammonium acetate for 14 days; 2: K + 1.0 g/L glucose for 14 days

70	*Rhodococcus* sp. IEGM 1379	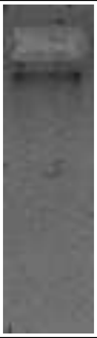	Metal beads (Ø=1 mm; quantity=10 beads; v=5 m/s; t=2 min)	RNeasy Mini Kit, Qiagen, Hilden, Germany	LB agar for 14 days
71	*Rhodococcus* sp. IEGM 1372	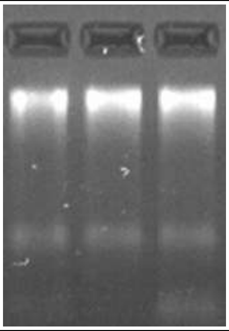	Metal beads (Ø=1 mm; quantity=10 beads; v=5 m/s; t=2 min)	RNeasy Mini Kit, Qiagen, Hilden, Germany	Agar K, propane : air 1 : 4 for 14 days
72	*Rhodococcus* sp. IEGM 1372	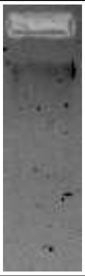	Metal beads (Ø=1 mm; quantity=10 beads; v=5 m/s; t=2 min)	RNeasy Mini Kit, Qiagen, Hilden, Germany	K without growth substrate for 14 days (oligotrophic conditions)
73	*Rhodococcus* sp. IEGM 1372	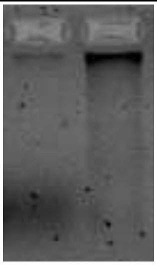	Metal beads (Ø=1 mm; quantity=10 beads; v=5 m/s; t=2 min)	RNeasy Mini Kit, Qiagen, Hilden, Germany	From left to right: 1: K + 20.0 g/L ammonium acetate for 14 days; 2: K + 1.0 g/L glucose for 14 days
74	*Rhodococcus* sp. IEGM 1381	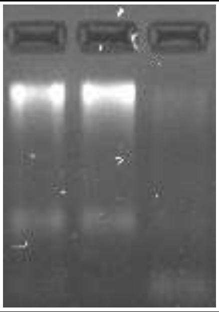	Metal beads (Ø=1 mm; quantity=10 beads; v=5 m/s; t=2 min)	RNeasy Mini Kit, Qiagen, Hilden, Germany	Agar K, propane : air 1 : 4 for 14 days

75	*Rhodococcus* sp. IEGM 1381	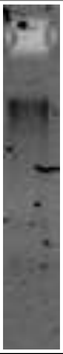	Metal beads (Ø=1 mm; quantity=10 beads; v=5 m/s; t=2 min)	RNeasy Mini Kit, Qiagen, Hilden, Germany	K without growth substrate for 14 days (oligotrophic conditions)
76	*Rhodococcus* sp. IEGM 1381	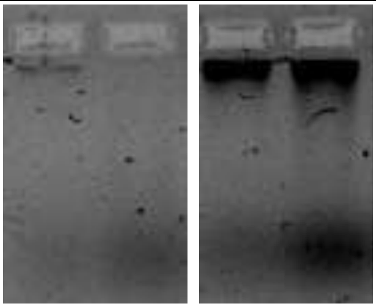	Metal beads (Ø=1 mm; quantity=10 beads; v=5 m/s; t=2 min)	RNeasy Mini Kit, Qiagen, Hilden, Germany	From left to right: 1,3: K + 1.0 g/L glucose for 14 days; 2,4: K + 20.0 g/L ammonium acetate for 14 days
77	*Rhodococcus* sp. IEGM 1404	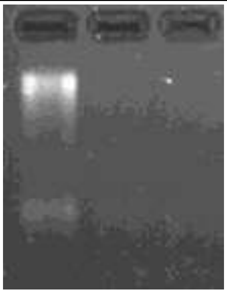	Metal beads (Ø=1 mm; quantity=10 beads; v=5 m/s; t=2 min)	RNeasy Mini Kit, Qiagen, Hilden, Germany	Agar K, propane : air 1 : 4 for 14 days
78	*Rhodococcus* sp. IEGM 1404	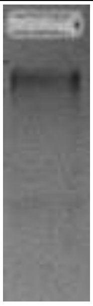	Metal beads (Ø=1 mm; quantity=10 beads; v=5 m/s; t=2 min)	RNeasy Mini Kit, Qiagen, Hilden, Germany	K without growth substrate for 14 days (oligotrophic conditions)
79	*Rhodococcus* sp. IEGM 1404	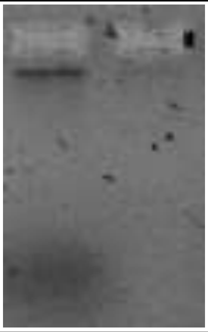	Metal beads (Ø=1 mm; quantity=10 beads; v=5 m/s; t=2 min)	RNeasy Mini Kit, Qiagen, Hilden, Germany	From left to right: 1: K + 1.0 g/L glucose for 14 days; 2: K + 20.0 g/L ammonium acetate for 14 days

80	*R. cerastii* IEGM 1243	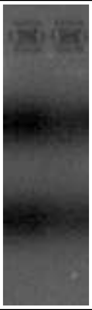	Metal beads (Ø=1 mm; quantity=10 beads; v=5 m/s; t=2 min)	RNA Solo, Evrogen, Russia (from left to right): 1: with DNase purification; 2: without DNase purification	LB for 48 h
81	*R. cerastii* IEGM 1243	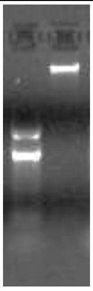	Lysozyme (40 mg/mL), 37°C for 1 h + 10% SDS	RNA Solo, Evrogen, Russia (from left to right): 1: with DNase purification; 2: without DNase purification	LB for 48 h
82	*R. rhodochrous* IEGM 757	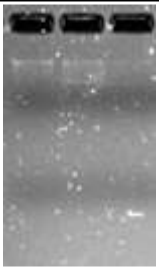	Without lysis	RNeasy Mini Kit, Qiagen, Hilden, Germany	From left to right: 1: RS + 0.1 g/L yeast extract for 48 h (resting cells); 2: RS + 0.1 g/L yeast extract + 1.0 g/L oleanolic acid for 48 h (resting cells); 3: RS + 0.1 g/L yeast extract + 1.0 g/L glycyrrhetinic acid for 48 h (resting cells)
83	*R. rhodochrous* IEGM 757	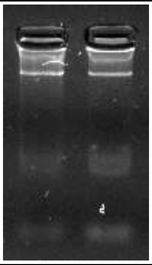	Lysozyme (40 mg/mL), 37°C for 1 h + 10% SDS	TRIzol™ LS Reagent, Invitrogen, Carlsbad, CA, USA	RS + 0.1 g/L yeast extract + 1.0 g/L oleanolic acid for 48 h (resting cells)
84	*R. ruber* IEGM 346	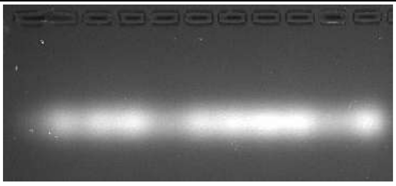	Metal beads (Ø=1 mm; quantity=10 beads; v=5 m/s; t=2 min)	RNeasy Mini Kit, Qiagen, Hilden, Germany	From left to right: 1-3: RS + 0.01 g/L glucose for 48 h; 4-6: RS + 0.01 g/L naproxen + 0.01 g/L glucose for 48 h; 7-9: RS + 0.01 g/L mixture of three NSAIDs (diclofenac, ibuprofen, naproxen) for 48 h;

					10-11: LB for 48 h
85	*R. ruber* IEGM 346	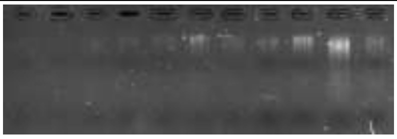	Metal beads (Ø=1 mm; quantity=10 beads; v=5 m/s; t=2 min)	RNeasy Mini Kit, Qiagen, Hilden, Germany	From left to right: 1-3: RS + 0.01 g/L glucose for 48 h; 4-6: RS + 0.01 g/L naproxen + 0.01 g/L glucose for 48 h; 7-9: RS + 0.01 g/L mixture of three NSAIDs (diclofenac, ibuprofen, naproxen) for 48 h; 10-11: LB for 48 h
86	*R. ruber* IEGM 1122	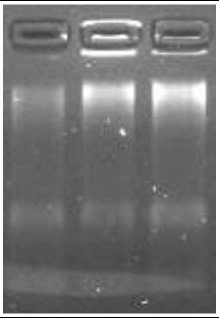	Metal beads (Ø=1 mm; quantity=10 beads; v=5 m/s; t=2 min)	RNeasy Mini Kit, Qiagen, Hilden, Germany	Agar K, propane : air 1 : 4 for 14 days
87	*R. ruber* IEGM 1122	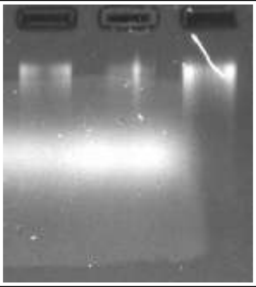	Metal beads (Ø=1 mm; quantity=10 beads; v=5 m/s; t=2 min)	RNeasy Mini Kit, Qiagen, Hilden, Germany	Agar K without growth substrate for 14 days (oligotrophic conditions)
88	*Rhodococcus* sp. IEGM 1307	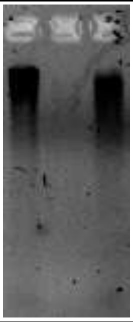	Metal beads (Ø=1 mm; quantity=10 beads; v=5 m/s; t=2 min)	RNeasy Mini Kit, Qiagen, Hilden, Germany	Agar K, propane : air 1 : 4 for 14 days
89	*Rhodococcus* sp. IEGM 1307	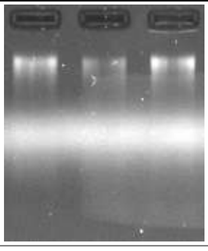	Metal beads (Ø=1 mm; quantity=10 beads; v=5 m/s; t=2 min)	RNeasy Mini Kit, Qiagen, Hilden, Germany	Agar K without growth substrate for 14 days (oligotrophic conditions)

90	*Rhodococcus* sp. IEGM 1341	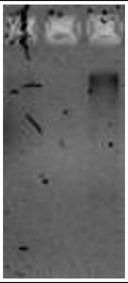	Metal beads (Ø=1 mm; quantity=10 beads; v=5 m/s; t=2 min)	RNeasy Mini Kit, Qiagen, Hilden, Germany	Agar K, propane : air 1 : 4 for 14 days
91	*Rhodococcus* sp. IEGM 1341	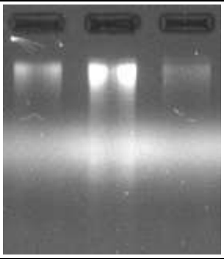	Metal beads (Ø=1 mm; quantity=10 beads; v=5 m/s; t=2 min)	RNeasy Mini Kit, Qiagen, Hilden, Germany	Agar K without growth substrate for 14 days (oligotrophic conditions)
92	*Rhodococcus* sp. IEGM 1351	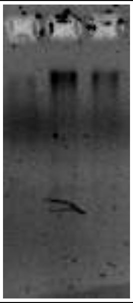	Metal beads (Ø=1 mm; quantity=10 beads; v=5 m/s; t=2 min)	RNeasy Mini Kit, Qiagen, Hilden, Germany	Agar K, propane : air 1 : 4 for 14 days
93	*Rhodococcus* sp. IEGM 1351	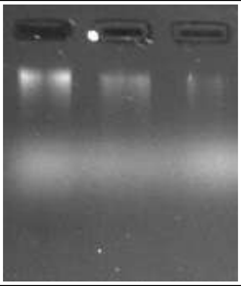	Metal beads (Ø=1 mm; quantity=10 beads; v=5 m/s; t=2 min)	RNeasy Mini Kit, Qiagen, Hilden, Germany	Agar K without growth substrate for 14 days (oligotrophic conditions)
94	*G. alkanivorans* IEGM 1277	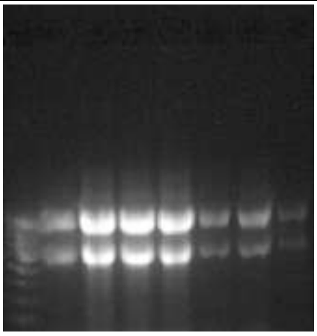	Lysozyme (40 mg/mL), 37°C for 1 h + 10% SDS	RNA Solo, Evrogen, Moscow, Russia	From left to right: 1: DNA ladder (250 bp–3 kb); 2-5: RS + 0.001 vol.% meloxicam + 0.1 vol.% *n*-hexadecane for 48 h; 6-7: RS + 0.1 vol.% *n*-hexadecane for 48 h; 8: LB for 48 h

95	*G. paraffinivorans* IEGM 735	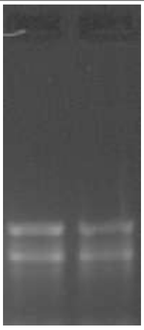	Lysozyme (40 mg/mL), 37°C for 1 h	RNA Solo, Evrogen, Moscow, Russia	From left to right: 1: LB for 96 h; 2: LB + 10.9 mg/L $HgCl_2$ for 96 h
96	*G. rubripertincta* IEGM 133	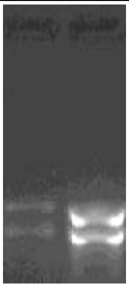	Lysozyme (40 mg/mL), 37°C for 1 h	RNA Solo, Evrogen, Moscow, Russia	From left to right: 1: LB for 96 h; 2: LB + 10.9 mg/L $HgCl_2$ for 96 h
97	*R. pyridinivorans* IEGM 639	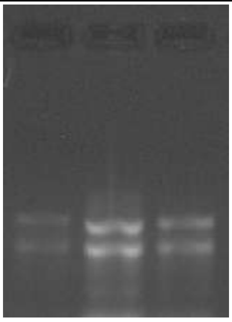	Lysozyme (40 mg/mL), 37°C for 1 h	RNA Solo, Evrogen, Moscow, Russia	From left to right: 1: LB for 96 h; 2: LB + 10.9 mg/L $HgCl_2$ for 96 h; 3: LB + 86.9 mg/L $HgCl_2$ for 96 h
98	*R. rhodochrous* IEGM 107	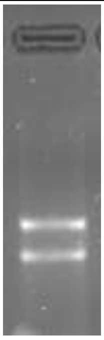	Lysozyme (40 mg/mL), 37°C for 1 h	RNA Solo, Evrogen, Moscow, Russia	RS + 0.025 vol.% (−)-isopulegol + 0.1 g/L yeast extract + 0.1 vol.% halloysite for 120 h (resting cells)
99	*R. rhodochrous* IEGM 1162	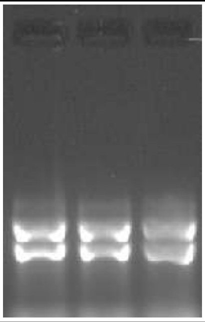	Lysozyme (40 mg/mL), 37°C for 1 h	RNA Solo, Evrogen, Moscow, Russia	From left to right: 1: LB for 96 h; 2: LB + 10.9 mg/L $HgCl_2$ for 96 h; 3: LB + 86.9 mg/L $HgCl_2$ for 96 h

100	*R. rhodochrous* IEGM 1360	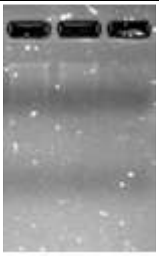	Without lysis	RNeasy Mini Kit, Qiagen, Hilden, Germany	From left to right: 1: RS + 0.1 g/L yeast extract for 48 h (resting cells); 2: RS + 1.0 g/L oleanolic acid + 0.1 g/L yeast extract for 48 h (resting cells); 3: RS + 1.0 g/L glycyrrhetinic acid + 0.1 g/L yeast extract for 48 h (resting cells)
101	*R. ruber* IEGM 439	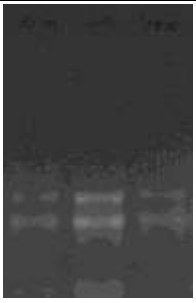	Lysozyme (40 mg/mL), 37°C for 1 h	RNA Solo, Evrogen, Moscow, Russia	From left to right: 1: LB for 96 h; 2: LB + 10.9 mg/L $HgCl_2$ for 96 h; 3: LB + 86.9 mg/L $HgCl_2$ for 96 h
102	*R. ruber* IEGM 560	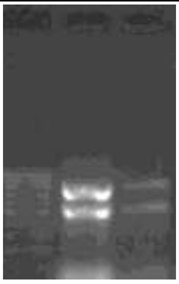	Lysozyme (40 mg/mL), 37°C for 1 h	RNA Solo, Evrogen, Moscow, Russia	From left to right: 1: DNA ladder (250 bp–3 kb); 2: LB + 10.9 mg/L $HgCl_2$ for 96 h; 3: LB + 86.9 mg/L $HgCl_2$ for 96 h

The reference electropherograms are shown. The lanes on the electropherograms correspond to different biological replicates unless otherwise stated. RNA Solo Kit was used with integrated DNase purification, unless otherwise stated. TRIzol™ LS and RNeasy Mini Kit were used without DNase treatment unless otherwise stated. Upper bands close to wells in electropherograms correspond to DNA. In some DNase-treated samples, the bands above the 23S rRNA band appear in electropherograms, and they correspond to pre-ribosomal RNA precursors (pre-rRNA), which are the primary transcripts of the rRNA operon [64]. The bands below the 16S rRNA band correspond to 5S rRNA, tRNA, or other small non-coding RNA.

Table S5. Reverse transcription reaction mixture preparation

Component	Volume
Total RNA	10 µl
10 × RT Mix	2 µl
HiScript III Enzyme Mix	2 µl
Random hexamers	1 µl
RNase-free ddH2O	5 µl
Total reaction volume	20 µl
Step1: Incubated for 15 min at 42 °C.	
Step 2: Incubated for 5 sec at 85 °C to inactivate reverse transcriptase after the reaction is over.	

Table S6. Specific primers for the target gene and the reference genes candidates of *R. ruber* IEGM 231

Gene	Gene annotation	Primers (FP – forward; RP – reverse)	Amplicon size, bp	PCR efficiency, %	R^2
16S rRNA	16S ribosomal RNA	FP: 5′-AGAGTTTGATCCTGGCTCAG-3′; RP: 5′-CGGTTACCTTGTTACGACTT-3′	1465	94.2	0.993
gyrA	DNA gyrase subunit A	FP: 5′-CGCGCCACAGCACCTCTAGCTC-3′; RP: 5′-GCAGGTGCTACTCGCGAGGCTG-3′	1550	93.5	0.991
gyrB	DNA gyrase subunit B	FP: 5′-GTCACTGATGCCACGAAGGAGGTA-3′; RP: 5′-TACTAGCGGACCTGGAGCAACAAG-3'	1494	94.8	0.994
dnaG	DNA primase	FP: 5′-GGGCGAATTTCAGACAGGGA-3′; RP: 5′-AATGTCTTCGCCGTGGTCTT-3′	1022	97.1	0.995
alkB	Alkane-1 monooxygenase	FP: 5′-GACGACGTCGGACATCG-3′; RP: 5′-CTGTGCTCCGTACTGCG-3′	1219	93.9	0,992

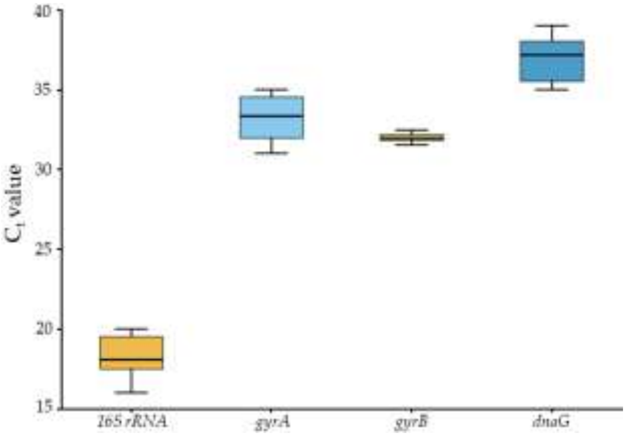

Figure S1. Distribution of C_t values for candidate reference genes. The line across the box depicts the median. The box indicates the 25th and 75th percentiles and the caps represent the maximum and minimum values.

	DeltaCT	BestKeeper	NormFinder	RefFinder
gyrA	2.00	2.00	2.31	3.63
dnaG	1.20	1.20	1.33	2.08
16S rRNA	1.18	1.18	1.42	2.52
gyrB	0.11	0.11	1.26	1.26

Figure S2. Heat map of expression stability of candidate genes for normalization of qRT-PCR data. Green corresponds to the highest expression stability, yellow corresponds to the average, and red corresponds to the lowest.

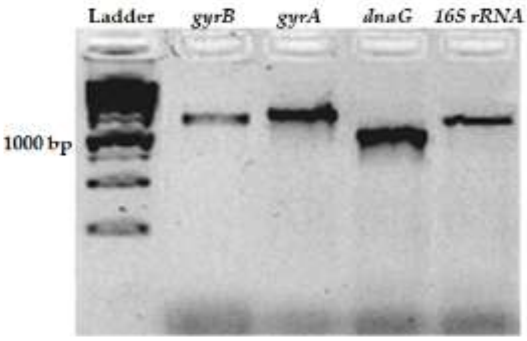

Figure S3. Electropherogram of PCR products of *R. ruber* IEGM 231 with specific primers for reference genes. The DNA ladder of the size from 250 to 3000 bp was used.

Table S7. Counting the relative expression ratio using Pfaffl method (SD – standard deviation; CV – coefficient of variation; R – relative expression ratio).

Gene	Type	C_t mean (n = 3)	SD	CV, %	ΔC_t	R
16S rRNA*	control	18.40	0.15	0.82	2.50	–
	sample	15.90	0.25	1.57		
gyrB	control	32.00	0.11	0.34	0.50	–
	sample	31.50	0.18	0.57		
*alkB*1	control	33.10	0.35	1.06	7.90	238.7**
	sample	25.20	0.20	0.79		

*The gene 16S rRNA showed a relatively high stability appropriate for the gene expression analysis that is in contrast to the revealed enhanced (on 29%) RNA yield in *Rhodococcus* cells grown in the presence of *n*-alkanes. This is plausibly related to the particular pair of growth conditions glucose-*n*-hexadecane (amounts of 16S rRNA in the C_3- or C_{10}-grown cells may be inappropriately higher compared to glucose, and normalization is required for each pair of growth conditions), as well as not only rRNA but mRNA contribute to the yield values. **The data are statistically reliable (p-value < 0.05).

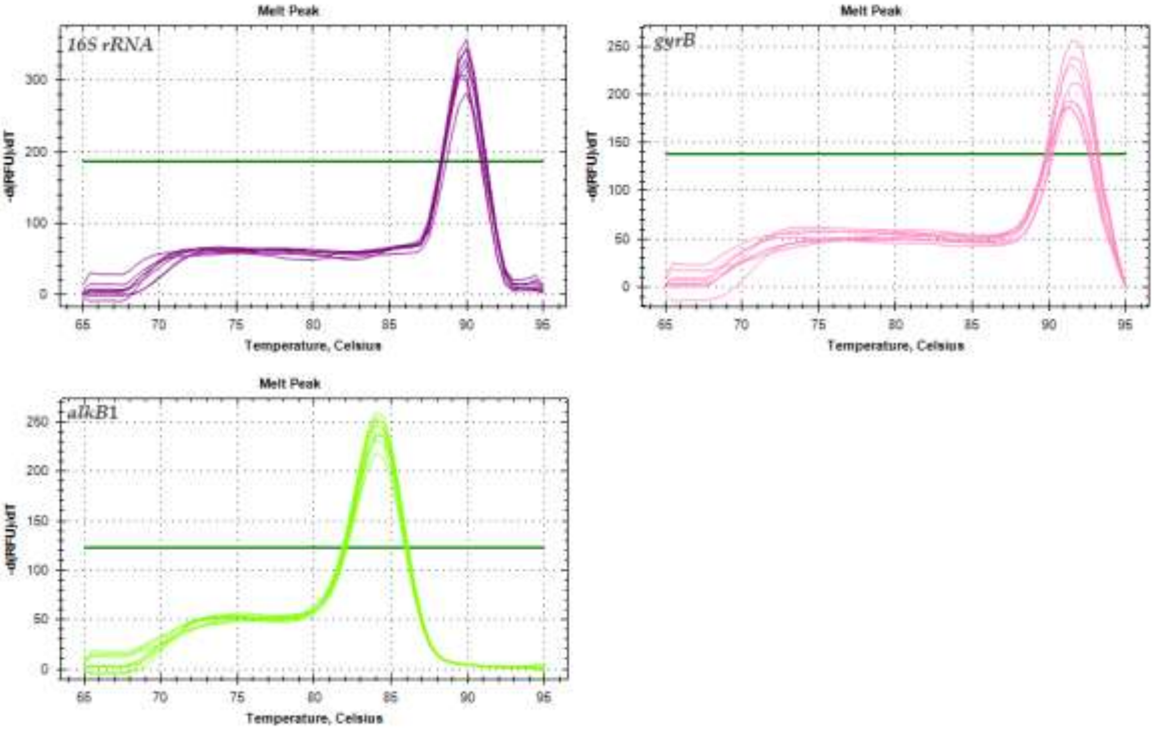

Figure S4. Melting curve analysis of *alkB* encoding gene.
